# A caged imidazopyrazinone for selective bioluminescence detection of labile extracellular copper(ii)†

**DOI:** 10.1039/d1sc07177g

**Published:** 2022-03-23

**Authors:** Justin J. O'Sullivan, Valentina Medici, Marie C. Heffern

**Affiliations:** Department of Chemistry, University of California Davis One Shields Drive Davis CA 95616 USA mcheffern@ucdavis.edu; Department of Internal Medicine, Division of Gastroenterology and Hepatology, University of California Davis 4150 V Street, PSSB Suite 3500 Sacramento CA 95817 USA

## Abstract

Copper is an essential redox-active metal that plays integral roles in biology ranging from enzymatic catalysis to mitochondrial respiration. However, if not adequately regulated, this redox activity has the potential to cause oxidative stress through the production of reactive oxygen species. Indeed, the dysregulation of copper has been associated with a variety of disease states including diabetes, neurodegenerative disorders, and multiple cancers. While increasing tools are being developed for illuminating labile intracellular copper pools and the trafficking pathways in which they are involved, significantly less attention has been given to the analogous extracellular labile pool. To address this gap, we have developed a bioluminescence-based imaging probe, picolinic ester caged-diphenylterazine (pic-DTZ) for monitoring labile, extracellular copper using a coelenterazine-like imidazopyrazinone and the genetically-engineered, marine-based luciferase, nanoluciferase. Unlike the more commonly-used firefly luciferase, nanoluciferase does not require ATP, allowing its application to the extracellular milieu. pic-DTZ demonstrates high metal and oxidation state selectivity for Cu(ii) in aqueous buffer as well as selectivity for labile pools over coordinatively inaccessible protein-bound Cu(ii). We demonstrate the potential of pic-DTZ as a diagnostic tool in human serum and plasma for copper-associated diseases. Additionally, we apply pic-DTZ to lend insight into the extracellular copper dynamic in anticancer treatments.

## Abstract 2

A caged imidazopyrazinone can detect reactive extracellular copper in mammalian systems using bioluminescence generated with the ATP-independent nanoluciferase enzyme.

## Graphical abstract

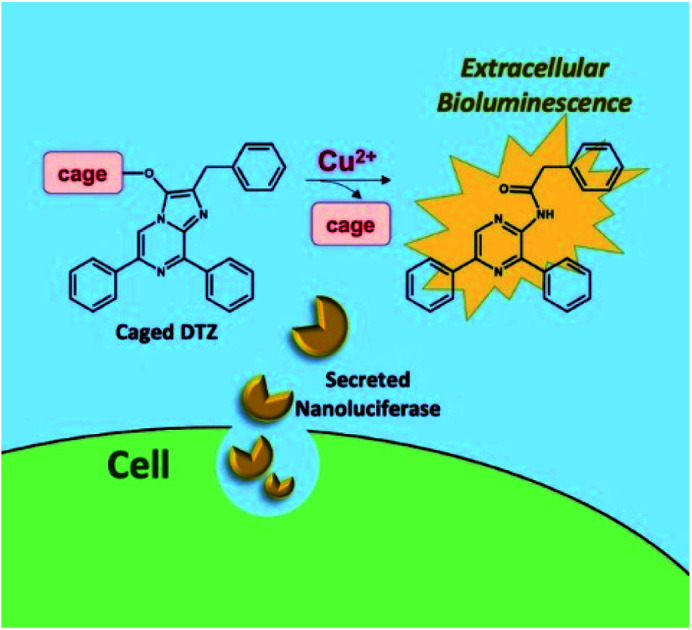

## Introduction

Copper is a trace micronutrient required for proper physiological function in all living organisms.^1,2^ Nature harnesses its accessible Cu(ii) and Cu(i) oxidation states for essential roles in enzymatic catalysis, mitochondrial respiration, and cell proliferation.^3–7^ Dysregulated copper biology has been associated with both aberrant redox activity and unwanted protein aggregation,^8^ implicating it in disease states that include obesity-associated metabolic disorders, neurodegeneration, and a variety of cancers.^9–11^ For this reason, biology has evolved complex systems to tightly regulate and traffic copper.

Increasing studies point to the importance in regulating not only the overall levels and redox states of biological copper, but its localization between the intra- and extracellular space. For example, Finney *et al.* used X-ray fluorescence spectroscopy to reveal large-scale relocalization and extracellular translocation of cellular copper during tumor angiogenesis.^12^ Widely studied yet still debated is the putative role of mislocalized extracellular copper in Alzheimer's disease and amyloid-beta aggregation.^13^ However, many of these studies are limited to structural studies with isolated proteins in buffer, snapshots of copper localization in fixed tissues and cells, or indirect measures *via* tracking of copper-dependent proteins and enzymes.^14–17^ The ability to target and monitor the copper pools in both the intracellular and extracellular milieu would provide valuable insight into copper dynamics in both normal physiological and pathological states.

Researchers have put forth powerful imaging probes for tracking intracellular copper that have illuminated the importance of the labile copper pool in dynamic cellular signaling.^18^ Distinct from the static copper pool, which is tightly-bound within protein pockets, the labile pool is loosely-bound to its ligands or have exchangeable coordination sites, allowing for exchange between biomolecules and low-molecular-weight chelates. Indeed, changes in the labile pool in extracellular fluids, such as blood and urine, have been observed in copper-associated disease states. For instance, in Wilson's disease (WD), a genetic disease resulting from pathogenic variants of the ATP7B copper transporter, serum samples exhibit elevation of non-ceruloplasmin-bound copper while total serum copper levels are not significantly affected.^19^ Acquiring knowledge to track both tissue and extracellular copper fluxes in cases such as this would have diagnostic potential and will shed light on the systemic manifestations of the disease.

Despite the advances in probe development for visualizing intracellular labile Cu(i) pools, tools for directly monitoring the labile, extracellular copper pool remain scarce.^17^ Extracellular copper measurements typically rely on either quantification of total copper levels by analytical techniques such as ICP-MS or monitoring blood levels of the major copper chaperone protein, ceruloplasmin.^20,21^ Though these methods can provide useful information particularly to total copper levels, they do not capture real-time changes in localization or cycling between the copper pools.^17^ To investigate the labile pool, researchers have paired the existing techniques with pre-analysis separation steps, such as chelation followed by size-exclusion enrichment.^22,23^ While these approaches have indeed expanded our understanding of the labile pool, they may perturb the native copper speciation.^24^ Thus, chemo-responsive imaging agents that directly target extracellular labile copper would preserve and inform on the copper dynamics outside of the cell and offer a platform for copper-associated diagnostics. An ideal probe would be selective for Cu(ii), the expected oxidation state of the labile pool in the extracellular space, target labile rather than tightly-bound pools, generate signal corresponding to extracellular rather than intracellular populations, and provide a turn-on a response, which can be a challenge due to the high fluorescence quenching ability of Cu(ii).^17^

**Scheme 1:**
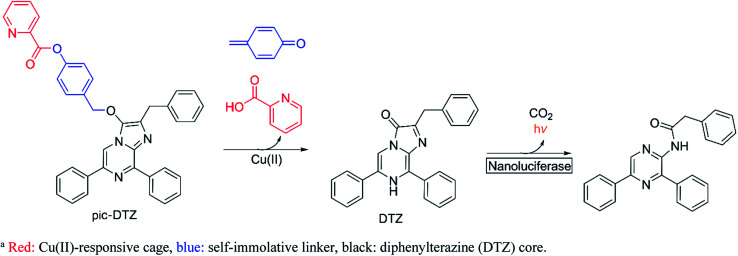
Design of pic-DTZ for Cu(ii)-responsive bioluminescence^*a*^.

Here we report the design, synthesis, and biological applications of a bioluminescent probe for monitoring labile, extracellular Cu(ii) pools with high selectivity and sensitivity. The picolinic ester-caged diphenylterazine (pic-DTZ) is a copper-responsive caged imidazopyrazinone probe that generates diphenylterazine (DTZ) upon ester hydrolysis by Cu(ii) for subsequent enzymatic conversion by the Nanoluciferase (Nluc) enzyme to produce light (Scheme 1). Extracellular targeting can be achieved by the selective localization or addition of Nluc. We demonstrate the selectivity of the probe for Cu(ii) over other relevant biological metals and for coordinatively unsaturated and reactive pools over tightly-bound copper. We show the potential of the probe as a diagnostic tool for serum and plasma copper levels, including its ability to distinguish elevated labile Cu(ii) levels in the plasma of individuals with WD relative to the plasma from healthy individuals. Finally, we employ the probe to directly assess changes in labile extracellular Cu(ii) levels in a breast cancer cell line treated with various anti-cancer agents that are known or reported to perturb intracellular copper trafficking pathways.

## Results and discussion

### Design and synthesis of pic-DTZ

**Scheme 2:**
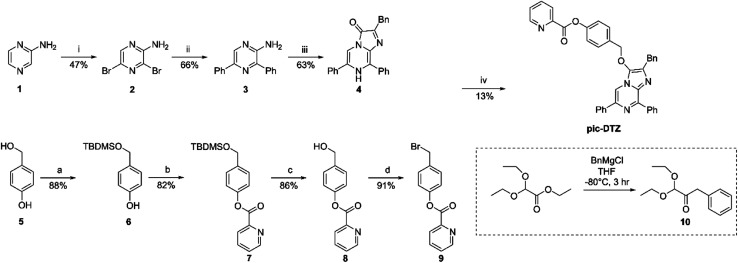
Synthesis of pic-DTZ^*a*^. ^*a*^Reagents and conditions: (i) NBS, CHCl_3_, 3 h; (ii) phenylboronic acid, Pd(PPh_3_)_4_, K_2_CO_3_, 1,4-dioxane, H_2_O, 80 °C, 12 h; (iii) 10, EtOH, H_2_O, cat. HCl, 80 °C, 12 h; (a) TBDMS-Cl, imidazole, DMF, r.t., 30 min; (b) 2-picolinic acid, EDCI, cat. DMAP, CH_2_Cl_2_. r.t., 3 h; (c) MeOH, cat. HCl, r.t., 30 min; (d) PBr_3_, THF, r.t., 2 h; (iv) 9, Cs_2_CO_3_, KI, MeCN, r.t., 12 h.

Bioluminescence, which involves the enzymatic conversion of luciferin substrates by luciferase enzymes to produce light, is an attractive imaging modality for its high signal-to-background ratio. Additionally, probes developed on this platform can be endowed with cell and tissue specificity through genetically encoding luciferase expression and localization.^25^ Caged luciferins are inert, chemically-modified derivatives of luciferin substrates that chemo-selectively react with the analyte of interest to restore the native luciferin which can then react with its cognate luciferase to produce a bioluminescent signal. Researchers have successfully developed a number of caged d-luciferins, the substrate of firefly luciferase.^18,26–28^ However, to the best of our knowledge, only three caged luciferins have been reported using a marine bioluminescent luciferin/luciferase pair.^29–31^ Marine bioluminescent systems all share the common imidazopyrazinone luciferin substrate, coelenterazine, that can react with a variety of marine luciferases such as *Renilla*, *Gaussia*, and the engineered Nluc.^32^ Unlike the firefly luciferase system, these systems do not require ATP as a cofactor, making them advantageous for extracellular applications. To generate Cu(ii)-responsive substrates, we exploited a previously reported Cu(ii)-dependent hydrolysis reaction using a picolinic ester design^33,34^ and incorporated a benzyl ether self-immolative linker^35^ to cage the imidazopyrazinone core. While we also explored the potential of a furan and thiophene ester cage, only the picolinic ester exhibited the desired metal-mediated hydrolysis (see ESI† discussion on probe development). We appended the picolinic ester cage to a series of imidazopyrazinone cores (Schemes 2 and S2†) and assessed their responsiveness with their cognate enzymes (Fig. S3†), ultimately finding the highest sensitivity with the caged DTZ, a synthetic coelenterazine analogue,^36^ paired with the engineered marine luciferase-derived Nluc.

### Reactivity and Cu(ii) selectivity of pic-DTZ in aqueous buffer with recombinant Nluc (rNluc)

**Fig. 1:**
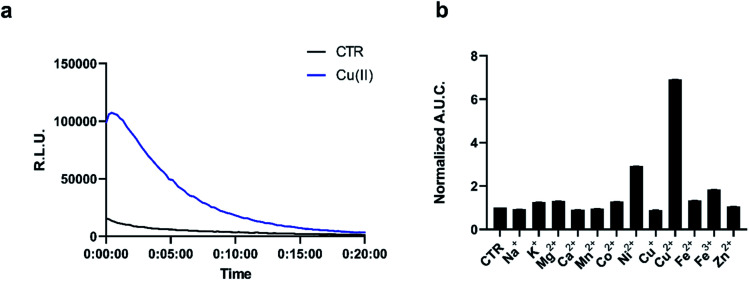
(a) Representative kinetic curve of luminescence of 5 μM pic-DTZ with or without the addition of 5 μM Cu(ii) in the presence of 120 nM rNluc. The control treatment (CTR) refers to the same conditions but with no metal salt added (b) calculated area under the curve for luminescence over 20 minutes of 5 μM pic-DTZ in the presence of biologically relevant d-block (5 μM) and s-block (1 mM) metals. Data points are normalized to signal of pic-DTZ without metal addition. Error bar denotes SEM, *n* = 3.

The chemo-selectivity of pic-DTZ was evaluated by reacting various analytes with the probe in aqueous buffer (DPBS, pH 7.4), then monitoring enzymatic conversion with recombinant Nluc (rNluc). When exposed to Cu(ii), pic-DTZ exhibits an immediate seven-fold increase in luminescence relative to control treatment with no metal (CTR), demonstrating efficient uncaging in the presence of free Cu(ii) (Fig. 1a). When monitoring the reactivity of the cage by ultraviolet-visible (UV-Vis) spectrophotometry, we observe a distinct change of the peak at 266 nm towards a spectrum with two absorbance maxima at 280 nm and 345 nm, suggesting hydrolysis of the picolinic ester (Fig. S2†). We further validated that Cu(ii)-mediated hydrolysis of pic-DTZ releases the parent DTZ by monitoring the reaction using liquid chromatography (Fig. S4†). In the presence of excess CuSO_4_, pic-DTZ is immediately hydrolyzed to DTZ, while in the presence of sub-stoichiometric amounts of CuSO_4_ (1 : 10 Cu(ii) : pic-DTZ) partial conversion to native DTZ is observed, albeit over four hours suggesting that Cu(ii) is not acting catalytically. Furthermore, a dose dependence is observed for pic-DTZ reactivity from 0 to 10 μM Cu(ii) until signal saturation is reached (Fig. S5†). Taken together, these results provide evidence for pic-DTZ's applicability towards monitoring low concentrations of copper. In line with previously suggested mechanisms of copper-induced hydrolysis, pic-DTZ turn-on likely results from Cu(ii) coordination to the pyridine nitrogen, with the carbonyl acting as a Lewis acid thereby increasing the susceptibility of the ester to nucleophilic attack.^37^

Next we evaluated the selectivity of pic-DTZ for Cu(ii) over other biologically-relevant metals including transition, alkali, and alkaline earth metals (Fig. 1b). The data show that pic-DTZ demonstrates selectivity for Cu(ii) over any of the other metals. Although a slight turn-on is observed in the presence of Ni(ii), serum levels of Ni(ii) are estimated to be between 4 nM to 0.8 μM, significantly lower than Cu(ii) at 16 μM.^17,38^ Additionally, a labile nickel pool has not yet been observed or characterized in mammalian systems. In addition to metal selectivity, pic-DTZ exhibits an oxidation-state specific response to Cu(ii) with no detectable turn-on in the presence of Cu(i). Lastly, we investigated the effect of pH on the efficiency of Cu(ii)-mediated uncaging. Because conversion from pic-DTZ to DTZ requires self-immolation of the phenolate generated upon hydrolysis of the picolinate we suspected that this would be a pH-sensitive process. We observed Cu(ii)-induced turn on at pH 6.2, 7.0, and 8.8 with increasing light output with increases in pH likely due to stabilization of the phenolate and subsequent self-immolation (Fig. S6†).

### Application of pic-DTZ to measure copper levels in human serum and plasma

**Fig. 2:**
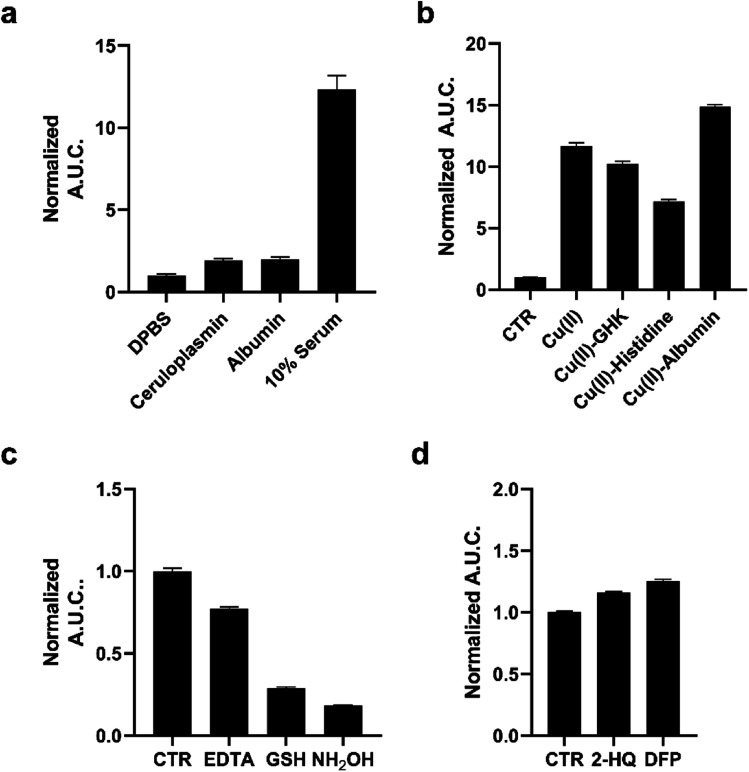
(a) Calculated area under the curve of luminescence taken over 20 minutes of 1 μM pic-DTZ in the presence of holo-ceruloplasmin (1000 mU mL^−1^), albumin (645 μM), or 10% pooled human serum in DPBS with 100 nM rNluc. (b) Calculated area under the curve of luminescence taken over 20 minutes of 1 μM pic-DTZ in the presence of Cu(ii) (10 μM), Cu(ii)–GHK (10 : 10 μM), Cu(ii)–histidine (10 : 10 μM), and Cu(ii)–albumin (100 : 100 μM) with 100 nM rNluc. (c) Calculated area under the curve of luminescence taken over 20 minutes of 1 μM pic-DTZ in 10% pooled human serum with EDTA (1 mM), GSH (200 μM), or NH_2_OH (200 μM) with 100 nM rNluc. (d) Calculated area under the curve of luminescence taken over 20 minutes of 1 μM pic-DTZ in 10% pooled human serum with esterase inhibitors 2-HQ and DFP (5 μM) with 100 nm rNluc. For (a–d) above error bars denote SEM, *n* = 3.

We next evaluated the capacity of pic-DTZ to detect labile Cu(ii) pools over tightly-bound populations in serum and plasma (Fig. 2A). Detection of Cu(ii) in biological fluids by optical methods are challenging due to the high background of these media from autofluorescence of its components in the UV/visible excitation and emission ranges.^17^ We reasoned that our system could overcome these challenges given the near-zero background signal of bioluminescence imaging.^39^ We first determined whether holo-ceruloplasmin (copper-bound form of the protein), the major carrier of Cu(ii) in the blood, would interfere with labile Cu(ii) detection by pic-DTZ. While ceruloplasmin carries anywhere from 50–90% of serum copper, the copper ions are tightly-bound within the protein pockets.^40^ When pic-DTZ is added to holo-ceruloplasmin at a physiologically-relevant concentration in the presence of rNluc, only a slight turn-on response was observed relative to the control (Fig. 2a), suggesting that pic-DTZ does not detect these static ceruloplasmin-bound copper pools. We also assessed the response of pic-DTZ to serum albumin. While Cu(ii)-bound albumin contributes to the labile extracellular pool, copper-free albumin has been shown to non-specifically oxidize coelenterazine, the core from which DTZ is derived.^41^ However, pic-DTZ again displayed only a slight turn-on response with copper-free albumin in the presence of rNluc, showing that this particular system is not susceptible to significant auto-oxidation by albumin.

We then applied pic-DTZ to a 10% solution of pooled human serum. A strong luminescence response was observed, suggesting that pic-DTZ can detect a copper pool that is distinct from that bound to ceruloplasmin. To characterize this pool, we performed a study with copper-loaded biological chelators to assess if pic-DTZ can turn-on in the presence of bound-copper species (Fig. 2b). We loaded the tripeptide GHK (10 μM), histidine (10 μM), and albumin (100 μM) with Cu(ii) in a 1 : 1 molar ratio and observed in all cases that pic-DTZ was still able to turn on suggesting that pic-DTZ is able to detect reactive copper species that are bound by these chaperones. Controls with GHK and histidine alone at this concentration showed little to no effect relative to the control (Fig. S6†). Although a slight turn-on is observed at the concentration of apo-albumin tested for this particular experiment (100 μM), the turn-on of pic-DTZ by holo-albumin at this same concentration is significantly higher (Fig. S8†).

To further validate that the observed signal in pooled human serum is resulting from reaction of pic-DTZ with Cu(ii) we used the chelator, EDTA, and two reductants, glutathione (GSH) and hydroxylamine (NH_2_OH), to reduce Cu(ii) to Cu(i), as Cu(i) would not readily hydrolyze the picolinate cage. As expected, EDTA and both reductants attenuated the turn-on response. Similar assays in buffer alone (in the absence of serum) had no effect on pic-DTZ activity, showing that EDTA and the reductants affect a pool of analytes that is present in serum (Fig. S9†). Lastly, as esterases are present in serum, we assessed whether such enzymes could interfere with pic-DTZ. We tested pic-DTZ's susceptibility to esterase activity by evaluating pic-DTZ signal in serum in the presence of diisopropylfluorophosphate (DFP) and 2-hydroxyquinoline (2-HQ), inhibitors for the two major plasma esterases, butyrylcholinesterase (BChE) and paraoxonase (PON1), respectively (Fig. 2d).^42^ The inhibitors did not decrease light output relative to the control serum, suggesting that plasma esterase activity does not interfere with pic-DTZ reactivity. Taken together, this data further suggests that the observed signal in serum is resulting from reaction of pic-DTZ with a pool of copper that is coordinatively unsaturated and accessible for reaction with pic-DTZ, which we term here as “reactive”.

**Fig. 3:**
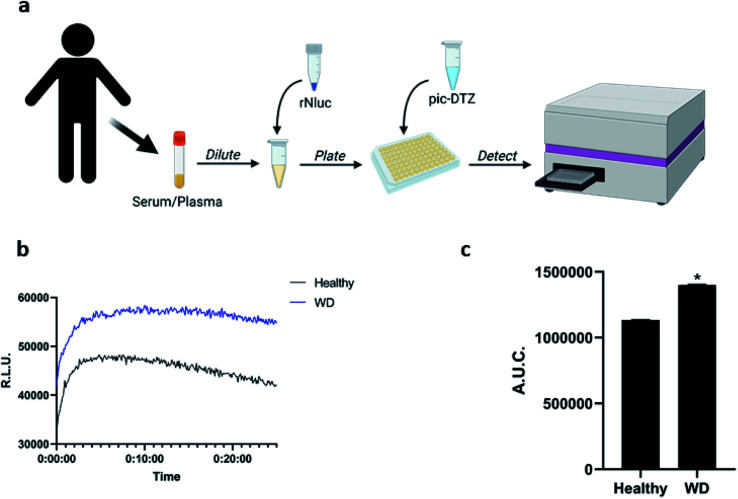
(a) Workflow for applying pic-DTZ for monitoring of labile copper status in human serum samples (figured created with BioRender.com) (b) representative kinetic curve of luminescence and (c) calculated area under the curve of luminescence over 20 minutes of plasma (diluted to 10% in DPBS) from healthy individuals or patients with Wilson's disease analyzed by addition of 1 μM pic-DTZ and 100 nM recombinant nanoluciferase. Error bar denotes SEM, *n* = 3. Statistical significance was assessed by calculating *p*-values using unpaired *t*-test, *p < 0.05.

Having observed the applicability of pic-DTZ for detecting reactive copper in serum, we assessed whether the probe could distinguish differences in the levels of this copper pool between individuals with WD and healthy individuals (Fig. 3). In WD, due to the absence of functional ATP7B, copper-free ceruloplasmin fails to mature properly in the hepatocytes and is rapidly degraded. Elevated levels of non-ceruloplasmin-bound copper is secreted from the hepatocytes into systemic circulation.^43^ The beneficial effects of chelation therapy in patients with WD suggest that this secreted copper is a labile pool. Serum ceruloplasmin levels are widely used as a screening and diagnostic test in WD, but this can lead to false negatives as most of these clinically-available assays do not distinguish between copper-bound and copper-free forms of the protein.^44^ Other diagnostic methods include determination of 24 hour urinary copper excretion and serum “free” copper levels. Serum “free” copper has classically been estimated *via* The Walshe's index by calculating the difference between total copper, determined by ICP-MS, and ceruloplasmin-bound copper.^45^ This index assumes that the measured ceruloplasmin is fully loaded with 6 copper atoms, and is therefore susceptible to over-estimation of copper-bound ceruloplasmin and underestimation of serum “free” copper. We reasoned that pic-DTZ could overcome these challenges by providing a direct means for detecting reactive copper(ii) in human plasma. Addition of pic-DTZ to the plasma of healthy controls and individuals with WD resulted in a significant (*P* < 0.05) elevation in bioluminescent response in the plasma of WD individuals (Fig. 3c). Notably, the serum albumin levels as well as overall protein levels between cohorts remained the same as would be expected suggesting that the observed changes in light output with pic-DTZ are indeed copper-dependent and not due to global changes (Fig. S10†). This demonstrates the probe's potential utility as a direct means of monitoring labile copper in serum and its potential as an alternative diagnostic tool for copper metabolism disorders.

### Monitoring extracellular copper(ii) in response to anti-cancer agents

**Fig. 4:**
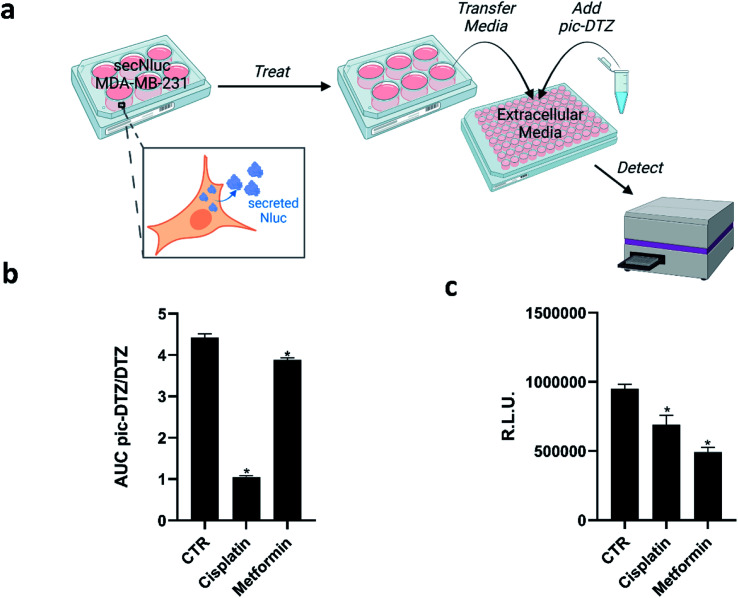
(a) Experimental workflow for applying pic-DTZ to monitoring extracellular copper in a breast cancer cell model (figure created with BioRender.com). (b) Calculated ratio of the area under the curve over 20 minutes of pic-DTZ : DTZ luminescence from secNluc MDA-MB-231 cells treated with cancer-associated agents reported to perturb copper metabolism: 30 μM cisplatin and 5 mM metformin. Error bars denote SEM, *n* = 3. Statistical significance was assessed by calculating *p*-values using unpaired *t*-test, *p < 0.05. (c) Cell viability in response to the cancer-associated agents in Fig. 4b as determined by CellTiter-Glo assay (Promega). Error bars denote SEM, (*n* = 4). Statistical significance was assessed by calculating *p*-values using unpaired *t*-test, *p < 0.05.

Copper has long been connected to cancer, with recent years identifying molecular mechanisms responsible for this interplay in a framework termed cuproplasia (copper-dependent cell proliferation).^46^ Stemming from these connections, chemicals that can chelate copper or modify copper trafficking pathways are emerging as potential cancer therapeutic agents.^47–51^ These copper-associated anticancer agents have been proposed to work *via* different mechanisms that perturb overall copper bioavailability to the cell, including extracellular chelation, intracellular depletion, and disrupting molecular pathways to alter metal distribution.^52^ However, studies that directly monitor the extracellular copper availability with these treatments remain sparse, and research has primarily focused on monitoring intracellular copper concomitant with tumor growth. We therefore explored the utility of pic-DTZ towards monitoring extracellular copper status of a metastatic breast cancer cell line, MDA-MB-231, stably expressing secreted Nluc (secNluc MDA-MB-231) in response to these agents (Fig. 4a).

Specifically, we assessed response to cisplatin, a well-established chemotherapy drug that has been shown to interfere with and hijack intracellular copper trafficking mechanisms;^53,54^ and metformin, an antidiabetic agent under consideration for cancer therapy and prevention, that has been shown to complex to and potentially rely on copper for its drug action.^55^ Although these agents have been associated with copper, little is known about their effects on extracellular copper populations. We treated cells with these agents using concentrations based on previous reports^55–57^ for 18 hours in a 96-well plate, followed by addition of either pic-DTZ or the parent DTZ. Luminescence was recorded and the area under the curve (AUC) was calculated. The data was analyzed using the ratios of the AUC pic-DTZ : DTZ to normalize any effects the treatments may have on cell viability or secNluc expression (Fig. 4b). We confirmed that neither compound interfered with pic-DTZ signal in the presence or absence of exogenous Cu(ii) (Fig. S11†). Both treatments showed significant decreases in extracellular copper-dependent signal but cisplatin treatment exhibited the most notable decrease in signal. Recent work by Akerfeldt *et al.* showed that the complex increases cytosolic copper availability.^48^ Our findings may complement this observation if this increase in intracellular copper stems from increased trafficking of copper from the extracellular to intracellular space. We further characterized the effects of the tested treatments by monitoring changes in protein expression of various copper-trafficking proteins *via* western blotting (Fig. S12†) and observed that cisplatin induced the strongest changes in protein expression relative to the untreated control in line with the observed drastic decrease in light output observed with pic-DTZ of cisplatin-treated cells. Interestingly, analysis of the cell media after treatment by ICP-MS showed no significant changes in total copper levels between the treated cells and untreated controls (Fig. S13†), suggesting that while total copper levels may not be significantly altered, the treatments may perturb a subpopulation of copper that is detectable with pic-DTZ.

The copper-responsive signals were distinct from bioluminescence responses from the same treatments monitored with CellTiter Glo (Fig. 4c). CellTiter Glo is a widely-used assay for measuring cell viability using the firefly luciferase system. The differential response between this bioluminescence-based cell viability assay and our pic-DTZ system shows the potential for bioluminescence multiplexing for high-throughput screening of copper-perturbing agents.

## Concluding remarks

We have demonstrated the design, synthesis, characterization, and biological applications of pic-DTZ, a new bioluminescent imaging probe for labile, extracellular Cu(ii) pools. To the best of our knowledge, the pic-DTZ/Nluc system is the first such caged imidazopyrazinone probe paired with the bright, thermostable Nluc as well as the first bioluminescent imaging probe for directly monitoring Cu(ii) in the extracellular space. pic-DTZ is metal- and redox state-specific for Cu(ii) in aqueous buffer with rNluc and can differentiate serum and plasma copper levels in clinical samples. Additionally, the probe system can be easily applied to a 96-well format for cell-based assaying of extracellular copper. We also show that pic-DTZ can be integrated into existing workflows and be multiplexed with existing firefly luciferase-based assays.

pic-DTZ offers unique advantages over traditionally explored methods of monitoring labile, extracellular copper. For example, pic-DTZ requires no excitation light source resulting in high signal-to-background ratios that are ideal for monitoring trace analytes like labile metal micronutrient pools. The reaction-based nature of pic-DTZ allows for signal amplification through the catalytic use of Cu(ii) relative to turn-on/turn-off probes. Paired with the locale-specificity afforded by genetically encoding Nluc, we envision that pic-DTZ has immense potential for application in live-animal models alongside emerging Nluc technologies.

## Experimental methods

### General methods

Reactions using moisture- or air-sensitive reagents were carried out in dried glassware under an inert N_2_ atmosphere. Dry solvents were all purchased from Sigma-Aldrich (St. Louis, MO) and used immediately. All commercially purchased chemicals were used as received without further purification. 2-Aminopyrazine was purchased from Oakwood Products, Inc. (Estill, SC); all other chemicals were purchased from Sigma-Aldrich. Silica Gel 60 F254 (precoated sheets, 200 μm thickness, MilliporeSigma) were used for analytical thin layer chromatography. Silica gel sorbent (230–400 mesh, grade 60, ThermoFisher) or aluminum oxide (neutral, Brockmann I, 50–200 μm, grade 60, Sigma-Aldrich) were used for column chromatography. ^1^H and ^13^C NMR spectra were collected at room temperature in CDCl_3_, DMSO-d_6_ or CD_2_Cl_2_ (Sigma-Aldrich) on a 400 or 800 MHz Bruker or 600 MHz Varian NMR spectrometer. All chemical shifts are reported as *δ* parts per million relative to the residual solvent peak at 7.26 (CDCl_3_), 2.50 (DMSO-d_6_), or 5.32 (CD_2_Cl_2_) for ^1^H and 77.16 (CDCl_3_), 39.52 (DMSO-d_6_) or 53.84 (CD_2_Cl_2_) for ^13^C. Multiplicities are reported as s (singlet), d (doublet), t (triplet), q (quartet), p (pentet), h (hextet), m (multiplet), dt (doublet of triplets), or br (broad). Electrospray ionization mass spectral analyses were performed using an LC-MSD system (Agilent Technologies 1260 Infinity II coupled with an Agilent Technologies InfinityLab LC/MSD).

### 2-Amino-3,5-dibromopyrazine, 2

2-Aminopyrazine (2.00 g, 21.92 mmol, 1 equiv.) and *N*-bromosuccinimide (7.86 g, 44.14 mmol, 2.1 equiv.) were dissolved in chloroform (45 mL). The reaction was allowed to stir at room temperature for three hours. It was then quenched with water and extracted with EtOAc (3 × 25 mL), washed with saturated sodium bicarbonate (5 × 100 mL), dried over Na_2_SO_4_, and concentrated *in vacuo* to yield 3 (2.51 g, 47%) as a pale brown solid. ^1^H NMR (400 MHz, CDCl_3_) *δ* 8.04 (s, 1H), 5.05 (br, 2H). Low-resolution mass spectrometry (LRMS) (*m*/*z*) [M + H]^+^ calculated for: C_4_H_4_Br_2_N_3_, 253.88; found, 253.8.

### 3,5-Diphenyl-2-aminopyrazine, 3

2-Amino-3,5-dibromopyrazine, 2, (812 mg, 3.22 mmol, 1 equiv.), phenylboronic acid (863 mg, 7.08 mmol, 2 equiv.), Pd(PPh_3_)_4_ (373 mg, 0.322 mmol, 0.1 equiv.), and K_2_CO_3_ (1.450 g, 10.5 mmol, 3.25 equiv.) were added to a flame-dried 250 mL round-bottom flask purged with N_2_. 1,4-Dioxane (65 mL) and water (15 mL) were added to the flask and the reaction mixture was heated to 80 °C. The reaction was allowed to stir for 12 hours before being quenched with water and extracted with DCM (3 × 25 mL), washed with brine, dried over, dried over Na_2_SO_4_, and concentrated *in vacuo*. The crude product was then purified by silica gel chromatography (60/40 heptanes/EtOAc). Combined product fractions were concentrated under reduced pressure to yield 4 (525 mg, 66%) as a green-brown solid. ^1^H NMR (400 MHz, CDCl_3_) *δ* 8.46 (s, 1H), 7.98 (d, 2H), 7.84 (d, 2H), 7.53 (t, 2H), 7.50–7.41 (m, 3H), 7.41–7.35 (m, 1H), 4.84 (s, 2H). Low-resolution mass spectrometry (LRMS) (*m*/*z*) [M + H]^+^ calculated for: C_16_H_14_N_3_, 248.12; found, 248.1.

### 1,1-Diethoxy-3-phenylpropan-2-one, 10

Ethyl diethoxyacetate (1.00 g, 5.68 mmol, 1 equiv.) was dissolved in THF (12 mL) and added to a flame-dried round-bottom flask purged with N_2_. It was then cooled to −78 °C (dry ice-acetone bath). To the mixture was added benzyl magnesium chloride (2 M solution in THF, 1.28 g, 4.26 mL, 1.5 equiv.) dropwise. The reaction was allowed to stir for two hours and then quenched with saturated NH_4_Cl solution. The reaction was then extracted with ethyl acetate (3 × 25 mL). The combined organic extract was washed with brine, dried over Na_2_SO_4_, and concentrated *in vacuo*. The crude product was used without further purification.

### Diphenylterazine, 4

3,5-Diphenyl-2-aminopyrazine, 3, (0.240 g, 0.889 mmol, 1 equiv.) and 1,1-diethoxy-3-phenylpropan-2-one, 10, were added to a N2-purged round-bottom flask followed by ethanol (11 mL), water (2 mL), and hydrochloric acid (0.2 mL). The reaction was then refluxed overnight. The reaction was then concentrated under reduced pressure and purified on an alumina gel column (gradient 0–10% MeOH in DCM) to afford 4 (211 mg, 63%) of pure product as a pale brown solid. ^1^H NMR (400 MHz, DMSO) *δ* 8.73 (s, 1H), 8.54 (s, 2H), 8.19 (d, 2H), 7.57–7.64 (m, 3H) 7.51–0.755 (t, 2H), 7.42–7.46 (t, 1H), 7.27–7.33 (m, 4H), 7.16–7.20 (t, 1H), 4.24 (s, 2H). Low-resolution mass spectrometry (LRMS) (*m*/*z*): [M + H]^+^ calculated for: C_25_H_20_N_3_O, 378.16; found, 378.1.

### 4-(((*tert*-Butyldimethylsilyl)oxy)methyl)phenol, 6

4-Hydroxybenzyl alcohol (1.00 g, 8.06 mmol, 1 equiv.), TBDMS-Cl (1.58 g, 10.47 mmol, 1.3 equiv.) and imidazole (1.31 g, 19.3 mmol, 2.6 equiv.) were added to a dry round-bottom flask and dissolved in DMF (5 mL) and allowed to stir under ambient conditions for 1 hour. The reaction was then quenched in water and extracted with ethyl acetate (3 × 25 mL). The combined ethyl acetate extracts were then washed with 0.1 M HCl (1 × 50 mL), sat. NaHCO_3_ (1 × 50 mL) and then brine (1 × 50 mL) and then dried over Na_2_SO_4_ and concentrated under reduced pressure. The crude product was then purified by silica gel column chromatography (50/50 hexanes/EtOAc) to afford 6 (1.7 g, 88% yield) as a pale-yellow oil. ^1^H NMR (400 MHz, CDCl_3_) *δ* 7.18 (d, 2H), 6.79 (d, 2H), 4.67 (s, 2H), 0.94 (s, 9H), 0.10 (s, 6H). ^13^C NMR (100 MHz, CDCl_3_) *δ* 155.01, 133.27, 127.99, 115.29, 77.48, 65.02, 26.12, 18.99, −5.04. Low-resolution mass spectrometry (LRMS) (*m*/*z*) [M + H]^+^ calculated for: C_13_H_23_O_2_Si, 239.15; found, 239.1.

### 4-(((*tert*-Butyldimethylsilyl)oxy)methyl)phenyl picolinate, 7

4-(((*tert*-butyldimethylsilyl)oxy)methyl)phenol, 6, (1.46 g, 5.71 mmol, 1 equiv.), 2-picolinic acid (0.878 g, 7.13 mmol, 1.25 equiv.), EDCI (1.37 g, 7.13 mmol, 1.25 equiv.), and DMAP (0.261 g, 2.14 mmol, 0.375 equiv.) were added to a dry round-bottom flask and dissolved in DCM (8 mL). The reaction was allowed to stir for three hours under ambient conditions until complete conversion was observed by LCMS. The reaction mixture was quenched in 100 mL of water and extracted with ethyl acetate (3 × 25 mL) and the combined ethyl acetate extracts were washed with brine, dried over Na_2_SO_4_, and then concentrated under reduced pressure. The crude product was purified by silica gel column chromatography (50/50 hexanes/EtOAc) to afford 7 (1.6 g, 82%) as a pale-yellow oil. ^1^H NMR (400 MHz, CDCl_3_) *δ* 8.2–8.5 (d, 1H), 8.26 (d, 1H), 7.88–7.92 (dt, 1H), 7.52–7.56 (m, 1H), 7.37–7.39 (d, 2H), 7.20–7.22 (d, 2H), 4.76 (s, 2H), 0.94 (s, 9H), 0.11 (s, 6H). ^13^C NMR (100 MHz, CDCl_3_) *δ* 164.00, 150.12, 149.73, 147.56, 139.38, 137.22, 127.41, 127.08, 125.87, 121.39, 120.15, 64.43, 25.97, 25.67, 18.43, −5.24. Low-resolution mass spectrometry (LRMS) (*m*/*z*): [M + H]^+^ calculated for: C_19_H_26_NO_3_Si, 344.17; found, 344.2.

### 4-(Hydroxymethyl)phenyl picolinate, 8

4-(((*tert*-Butyldimethylsilyl)oxy)methyl)phenyl picolinate, 7, (1.6 g, 4.66 mmol) was dissolved in 0.1% HCl in methanol (10 mL) and was allowed to stir under ambient conditions for 30 minutes. The reaction mixture was then quenched with 100 mL of water and extracted into ethyl acetate (3 × 25 mL), washed with brine, dried over Na_2_SO_4_ and concentrated under reduced pressure. The product 8 (920 mg, 86%) was obtained as a white powder and used without further purification. ^1^H NMR (400 MHz, CDCl_3_) *δ* 8.74–8.76 (d, 1H), 8.22–8.24 (d, 1H), 7.86–7.90 (dt, 1H), 7.50–7.54 (m, 1H), 7.36–7.38 (d, 2H), 7.16–7.18 (d, 2H), 4.65 (s, 2H). ^13^C NMR (100 MHz, CDCl_3_) *δ* 163.93, 156.27, 150.05, 147.29, 139.17, 137.43, 128.79, 128.12, 127.62, 125.95, 121.66, 115.55, 77.16, 63.93. Low-resolution mass spectrometry (LRMS) (*m*/*z*): [M + H]^+^ calculated for: C_13_H_12_NO_3_, 230.08; found, 230.1.

### 4-(Bromomethyl)phenyl picolinate, 9

4-(Hydroxymethyl)phenyl picolinate, 8, (856 mg, 3.73 mmol, 1 equiv.) was added to a flame-dried round-bottom flask and the flask was purged with N_2_. DCM (7 mL) was then added to the flask followed by PBr_3_ (1.3 g, 4.85 mmol, 1.2 equiv.). The reaction was allowed to stir at room temperature under a N_2_ atmosphere for two hours and was then quenched with water (100 mL) and extracted into ethyl acetate (3 × 25 mL). The combined ethyl acetate extract was then washed with brine and dried over Na_2_SO_4_ and then concentrated under reduced pressure. The crude product was then purified by silica gel column chromatography (25/75 petroleum ether/ethyl acetate) to obtain 9 (990 mg, 91%) as a white solid. ^1^H NMR (400 MHz, CDCl_3_) *δ* 8.80–8.82 (d, 1H), 8.22–8.24 (d, 1H), 7.86–7.90 (dt, 1H), 7.50–7.54 (m, 1H), 7.42–7.44 (d, 2H), 7.20–7.22 (d, 2H), 4.48 (s, 2H). ^13^C NMR (100 MHz, CDCl_3_) *δ* 163.71, 150.77, 150.17, 147.24, 137.33, 135.70, 130.36, 127.60, 125.96, 122.08, 32.71. Low-resolution mass spectrometry (LRMS) (*m*/*z*): [M + H]^+^ calculated for C_13_H_11_BrNO_2_, 292.00; found 292.0.

### pic-DTZ

Diphenylterazine, 4, (72 mg, 0.19 mmol, 1 equiv.), 4-(bromomethyl)phenyl picolinate, 9, (112 mg, 0.38 mmol, 2 equiv.), Cs_2_CO_3_ (25 mg, 0.076 mmol, 0.4 equiv.), and KI (35 mg, 0.21 mmol, 1.1 equiv.) were added to a flame-dried round-bottom flask that was then purged with N_2_. The reagents were then dissolved in anhydrous acetonitrile (2 mL) and the reaction was allowed to stir overnight. The reaction mixture was subsequently quenched in 20 mL of water and extracted in DCM (3 × 10 mL). The combined DCM extract was then washed with brine, dried over Na_2_SO_4_, and concentrated under reduced pressure. The crude product was then purified by reverse-phase HPLC (gradient, 70%:30% H_2_O/MeCN to 100% MeCN over 50 min on a T3 Atlantis column (Waters) and dried under reduced pressure to obtain pic-DTZ (14 mg, 13% yield) as a brown-red solid. ^1^H NMR (600 MHz, CD_2_Cl_2_) *δ* 8.86–8.88 (d, 2H), 8.79–8.81 (d, 1H), 8.22–8.24 (d, 1H), 8.09 (s, 1H), 8.01–8.03 (d, 2H), 7.91–7.94 (t, 1H), 7.49–7.58 (m, 6H), 7.36–7.44 (m, 5H), 7.27–7.32 (m, 4H), 7.20–7.23 (t, 1H), 5.08 (s, 2H), 4.18 (s, 2H). ^13^C NMR (150 MHz, CD_2_Cl_2_) *δ* 164.02, 151.92, 150.49, 147.68, 139.79, 138.37, 137.59, 137.39, 137.29, 136.73, 134.19, 134.16, 132.36, 130.59, 130.50, 130.14, 129.17, 128.90, 128.83, 128.55, 127.91, 126.76, 126.53, 126.15, 122.54, 109.89, 76.99, 33.82. Low-resolution mass spectrometry (LRMS) (*m*/*z*): [M + H]^+^ calculated for: C_38_H_29_N_4_O_3_, 589.22; found, 589.2.

### *In vitro* luminescence assays

Milli-Q water (18.2 MΩ) was used to prepare all aqueous solutions. Metal ion solutions were prepared to 15 μM in water (MgCl_2_, NaCl, KCl, CaCl_2_, MnCl_2_, (NH_4_)_2_Fe(SO_4_)·6H_2_O, FeCl_3_, CoCl_2_, NiCl_2_, Cu(MeCN)_4_(PF_6_), CuCl_2_ and ZnCl_2_). A 15 μM solution of pic-DTZ was prepared by initially dissolving pic-DTZ in 5% pure ethanol of the total volume and bringing it up in DPBS (Gibco) at pH 7.4 to a final concentration of 15 μM. A 120 nM solution of rNluc was prepared by adding 4 μL of 0.4 mg mL^−1^ stock solution (Promega, Nano-Glo Assay Kit) into 699 μL of DPBS at pH 7.4. 25 μL of rNluc solution was added to the wells of a white, opaque, flat-bottom 96-well plate followed by 25 μL of metal solution. Finally, pic-DTZ (25 μL) was added to all the wells using a multi-channel pipette and mixed well. The bioluminescent signal was immediately measured using a Molecular Devices SpectraMax i3x plate reader at 37 °C for 1 hour. For *in vitro* assays using albumin, ceruloplasmin and pooled human serum, a 645 μM solution of albumin (Fatty Acid Free, ThermoFisher) was prepared in DPBS, a 1000 mU mL^−1^ solution of ceruloplasmin was prepared from a stock solution (ceruloplasmin colorimetric activity kit, ThermoFisher), and a 10% pooled human serum solution was prepared in DPBS from a stock solution (Sigma-Aldrich). A 1 μM solution of pic-DTZ was prepared in 5% pure ethanol in DPBS from a frozen aliquot and a 100 nM solution of rNluc was prepared in DPBS from a frozen stock solution (0.4 mg mL^−1^, Promega, Nano-glo assay kit). For experiments using EDTA, GSH, and NH_2_OH (Sigma-Aldrich) solutions were prepared in DPBS (pH 7.4). For experiments using GHK (Bachem), histidine (Sigma-Aldrich) and albumin (Sigma-Aldrich), stock solutions were prepared in water with or without CuSO_4_ and allowed to incubate for 15 minutes before measuring luminescence. For experiments using 2-HQ and DFP (Sigma-Aldrich), stock solutions were prepared in ethanol and were added to 10% serum to a final concentration of 5 μM (ethanol final concentration was less than 5%) and incubated for 15 minutes before reading the luminescence. When reporting area under the curve, these values were calculated using GraphPad Prism software.

### Bioluminescent response to copper in plasma

Human plasma samples from healthy individuals (*n* = 3) and individuals with Wilson's disease (*n* = 3) were obtained by Dr Valentina Medici (UC Davis). All subjects provided written informed consent prior to participation following the Declaration of Helsinki. The protocol was approved by UCD Institutional Review Board (protocol # 818454). A 10% sample of plasma for each condition was prepared in DPBS and rNluc (Promega, 0.4 mg mL^−1^) from a frozen stock solution was added to obtain a final concentration of 100 nM enzyme. 100 μL of each sample was added into wells of a 96-well, white, flat-bottom opaque plate and 1 μL of a 100 μM pic-DTZ (5% pure ethanol in DPBS) solution was added to each well using a multi-channel pipette. Luminescence was immediately measured as described previously.

### Cell culture procedures

MDA-MB-231 cells stably expressing secreted nanoluciferase (secNluc MDA-MB-231) were a kind gift from Drs Gary and Kathy Luker (University of Michigan). Cells were maintained in Dulbecco's modified medium (DMEM) supplemented with 10% fetal bovine serum (FBS) (Gibco) 1× penicillin–streptomycin (Corning), 2 mM l-glutamine (Gibco), 1 mM sodium pyruvate (Gibco) at 37 °C and 5% CO_2_.

### Bioluminescent response to copper in secNluc MDA-MB-231 cells

Cells were plated at 10 000 cells per well in a 96-well, white, opaque flat-bottom plate. Eight hours later, the media was removed and the cells were washed with pre-warmed (37 °C) DPBS and 100 μL of OptiMem (Gibco) was added to each well. A 3 mM solution of cisplatin and 500 mM solution of metformin were prepared in water. 1 μL of these solutions were added to the wells for final concentrations of 30 μM cisplatin and 5 mM metformin. At the 18 hour time point solutions of 100 μM pic-DTZ and 100 μM DTZ (5% pure ethanol in DPBS) were prepared and 1 μL was added per well per treatment (*n* = 3). Luminescence was immediately recorded as previously described. The area under the curve was calculated using GraphPad Prism software and the ratio of the A. U. C. from pic-DTZ to DTZ was calculated.

## Data availability

The datasets supporting this article have been uploaded as part of the ESI.†

## Author contributions

J. J. O. and M. C. H. designed all experiments. J. J. O. performed all experiments and data collection. J. J. O. and M. C. H. analyzed the data. V. M. collected and provided the human serum samples. J. J. O. took the lead in writing the manuscript, and J. J. O. and M. C. H. wrote and edited the manuscript with input from V. M. M. C. H. conceived the study and was the major supervisor on overall direction of the study.

## Conflicts of interest

The authors report no conflicts of interest.

## Supplementary Material

SC-013-D1SC07177G-s001
