# Correction: A caged imidazopyrazinone for selective bioluminescence detection of labile extracellular copper(ii)

**DOI:** 10.1039/d2sc90085h

**Published:** 2022-05-03

**Authors:** Justin J. O’Sullivan, Valentina Medici, Marie C. Heffern

**Affiliations:** Department of Chemistry, University of California Davis One Shields Drive Davis CA 95616 USA mcheffern@ucdavis.edu; Department of Internal Medicine, Division of Gastroenterology and Hepatology, University of California Davis 4150 V Street, PSSB Suite 3500 Sacramento CA 95817 USA

## Abstract

Correction for ‘A caged imidazopyrazinone for selective bioluminescence detection of labile extracellular copper(ii)’ by Justin J. O’Sullivan *et al.*, *Chem. Sci.*, 2022, https://doi.org/10.1039/D1SC07177G.

The authors regret that a key organisation was omitted from the Acknowledgements section of their article. The correct Acknowledgement should read as follows:

This work was supported by the National Institute of Health (NIH MIRA 5R35GM133684-02 and NIH DK104770), the National Science Foundation (NSF CAREER 2048265). We also thank the Hartwell Foundation for their generous support for M. C. H. as a Hartwell Individual Biomedical Investigator, as well as the UC Davis CAMPOS Program and the University of California’s Presidential Postdoctoral Fellowship Program for their support of M. C. H. as a CAMPOS Faculty Fellow and former UC President’s Postdoctoral Fellow, respectively. This work was also supported in part by gift funds from the UC Davis Comprehensive Cancer Center. We thank Dr’s Gary and Kathy Luker (University of Michigan) for gifting MDA-MB-231 cell lines stably expressing secreted nanoluciferase. We also thank Joseph AbouAyash and Adam Hillaire for their support in the synthesis of precursors and the entire Heffern lab group for their support.

The Royal Society of Chemistry apologises for these errors and any consequent inconvenience to authors and readers.

## Supplementary Material

