# Supplementary material for: A caged imidazopyrazinone for selective bioluminescence detection of labile extracellular copper(ii)
Source: Chem Sci. 2022 Mar 23;13(15):4352–63. doi: 10.1039/d1sc07177g (PMC9006956; doi:10.1039/d1sc07177g)
Supplement: SC-013-D1SC07177G-s001 [file SC-013-D1SC07177G-s001.pdf]

Electronic Supplementary Material (ESI) for Chemical Science.
This journal is © The Royal Society of Chemistry 2022

Supporting Information for

A Caged Imidazopyrazinone for Selective Bioluminescence Detection of Labile Extracellular Copper(II)

Justin J. O'Sullivan, Valentina Medici, Marie C. Heffern

Table of Contents

Supplementary Discussion on Probe Development (including Figures S1-S3)

Additional Supplementary Figures (Figure S4 - S12)

Supplementary Materials and Methods

Synthesis of heterocyclic ester cages

Synthesis of pic-CTZ400a

Synthesis of pic-CTZ

Evaluation of pH responsiveness of pic-DTZ

ICP-MS analysis of copper in cell media

Cell Stimulations and Western Blot Analysis

Monitoring intracellular copper in MDA-MB-231 cells expressing cytosolic Nluc

NMR Spectra

- *3,5-dibromo-2-aminopyrazine, 2*
- *3,5-diphenyl-2-aminopyrazine, 3*
- *Diphenylterazine, 4*
- *4-(((tert-butyldimethylsilyl)oxy)methyl)phenol, 6*
- *4-(((tert-butyldimethylsilyl)oxy)methyl)phenyl picolinate, 7*
- *4-(hydroxymethyl)phenyl picolinate, 8*
- *4-(bromomethyl)phenyl picolinate, 9*
- *Pic-DTZ*
- *Thiophene Ester, 7a*
- *Furan ester, 7b*
- *3-benzyl-5-bromopyrazin-2-amine, 3b*
- *3-benzyl-5-phenylpyrazin-2-amine, 4b*
- *CTZ400a*
- *Pic-CTZ400a*
- *Pic-CTZ*

Supplementary Discussion 1: Selection of the substrate and chemoselective cage

Selection of imidazopyrazinone cores: To generate a copper-responsive bioluminescent agent that is compatible with the extracellular environment, we turned to agents derived from the marine bioluminescent systems as they operate independent of ATP, in contrast with the firefly luciferase/luciferin pair which requires ATP as a cofactor. As ATP is in low abundance in the extracellular space, eliminating ATP dependence is for signal sensitivity. Among the marine luciferases and its derivatives, both the naturally-occurring *Gaussia* luciferase and the engineered Nanoluciferase are significantly brighter than firefly luciferase by about 1000 times and 100 times respectively.[1,2] This feature is especially useful when developing probes for analytes that are present in trace amounts like the labile copper pool. Additionally both of these luciferases have been applied to extracellular applications using secreted forms of the enzymes.[3] One the one hand, the intensely bright, *Gaussia* luciferase has extremely low substrate tolerance, only accepting native coelenterazine.[3] On the other hand, a variety of coelenterazine derivatives have been synthesized that are accepted by Nanoluciferase in addition to its engineered substrate, furimazine.[4] We thus explored three different imidazopyrazinone-type compounds as cores for our probe: native coelenterazine (CTZ), coelenterazine 400a (CTZ400a) and diphenylterazine (DTZ) (Figure S1).

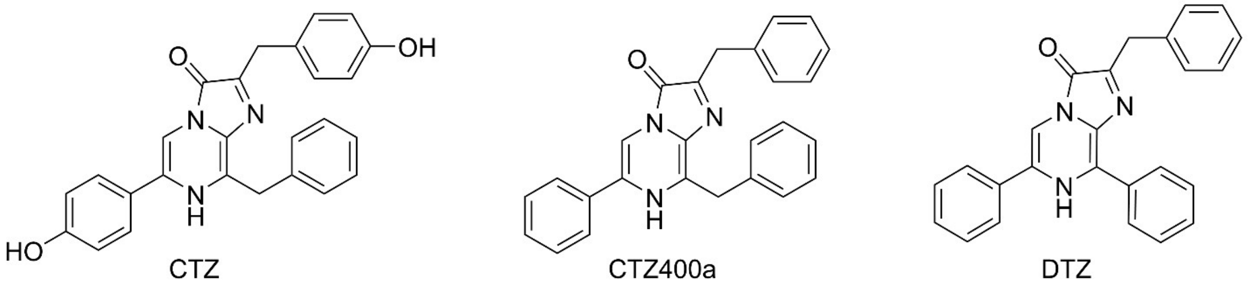

Figure S1. Chemical structures of coelenterazine, coelenterazine 400a, and diphenylterazine.

Selection of picolinic ester cage: For our cage design we adapted a previously-reported picolinic ester susceptible to hydrolysis by Cu(II).[5] We also explored the potential for other heterocyclic esters, furan and thiophene, to undergo metal mediated hydrolysis. The three heterocyclic ester cage precursors were synthesized as shown in Supplementary Scheme 1 (see scheme 2 in the main text for the synthesis of the pyridine ester cage **7**) and were subsequently tested for their response to metals by UV-Vis spectroscopy (Figure S2).

Scheme S1. Synthesis of heterocyclic ester cages.[a]

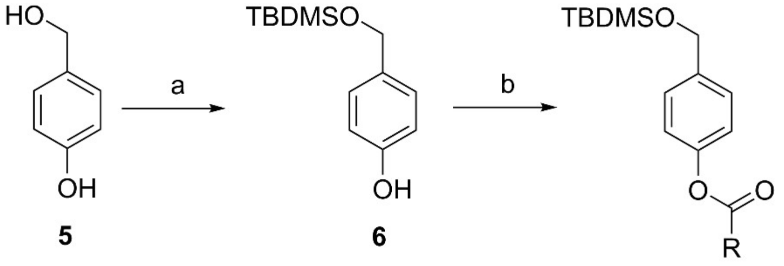

7a: R = 2-thiophene
7b: R = 2-furan

a. Reagents and conditions: (a) TBDMS-Cl, imidazole, DMF, r.t., 30 min; (b) furan-2-carbonyl chloride or thiophene-2-carbonyl chloride, anhyd. pyridine/DMF, r.t., 2 hours.

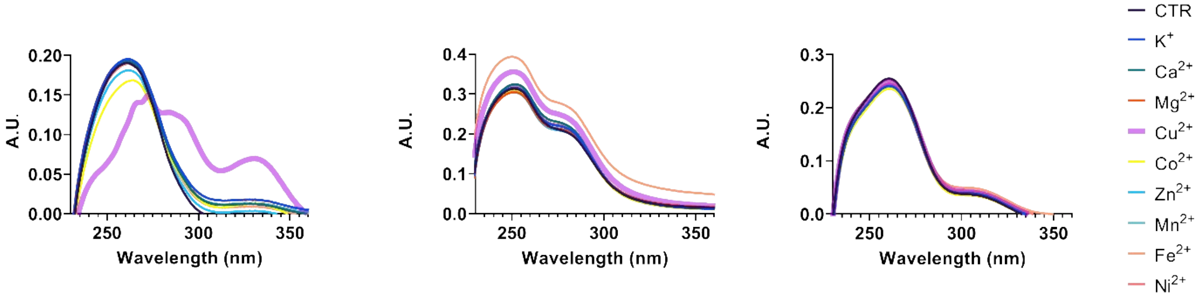

Figure S2. UV-Vis spectra of 10 µM (a) pyridine ester **7** (b) thiophene ester **7a,** and (c) furan ester **7b** in 15 mM MOPS buffer at pH 7.4 in the presence of 25 µM metal salts or a no-metal control (CTR). The marked shift in the spectra of the pyridine ester suggests hydrolysis in the presence of Cu^{2+}; while no distinct reactions are observed with the other two esters.

Consistent with the previous literature, we observed a distinct shift in the spectra of the picolinic ester upon addition of Cu(II) suggesting that the proposed Cu(II)-mediated hydrolysis occurs. However, we saw no changes to the spectra of either the thiophene or the furan ester upon the addition of any of the tested metals. With this information in hand, we proceeded with synthesis of the picolinic ester-caged imidazopyrazinones using the aforementioned cores (Scheme 2). It is well-known that these coelenterazine-like compounds readily degrade at ambient temperatures and in the presence of air likely due to the anti-aromatic nature of the lower pyrazine-like ring.To overcome this, researchers have been able to stabilize these compounds while maintaining bioluminescent activity by nucleophilic addition to the carbonyl thereby achieving complete aromaticity of the core.[6] Thus, we chose to conjugate the picolinic ester cage at the carbonyl position. For chemical compatibility, we employed a benzyl ether self-immolative linker.[7]

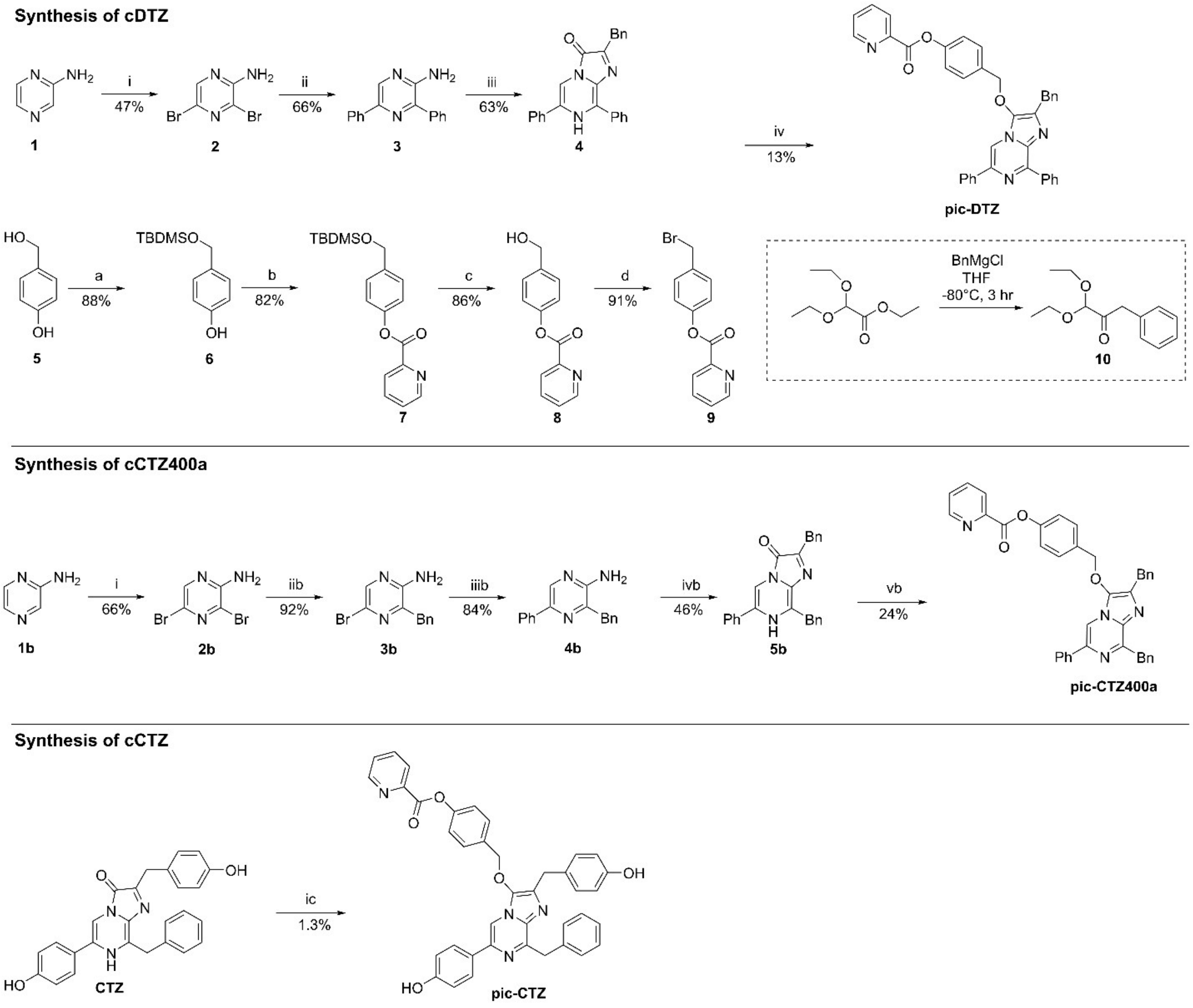

Scheme S2. Synthesis of pic-DTZ reagents and conditions: (i) NBS, $CHCl_3$, 3 h; (ii) Phenylyboronic acid, $Pd(PPh_3)_4$, K_2CO_3, Dioxane, H_2O, 80°C, 12 h; (iii) **10**, EtOH, H_2O, cat. HCl, 80°C, 12 h; (a) TBDMS-Cl, imidazole, DMF, r.t., 30 min; (b) 2-picolinic acid, EDCI, cat. DMAP, CH_2Cl_2. r.t., 3 h; (c) MeOH, cat. HCl, r.t., 30 min; (d) PBr_3, THF, r.t., 2 h; (iv) **9**, Cs_2CO_3, KI, MeCN, r.t., 12 h. **Synthesis of pic-CTZ400a reagents and conditions:** (iib) BnBr, Zn, I_2, $Pd(Cl)_2(PPh3)_2$, r.t. 12 h; (iiib) Phenylyboronic acid, $Pd(PPh_3)_4$, K_2CO_3, Dioxane, H_2O, 80°C, 12 h; (ivb) **10**, EtOH, H_2O, cat. HCl, 80°C, 12 h; (vb) MeOH, cat. HCl, r.t., 30 min. **Synthesis of pic-CTZ reagents and conditions:** (ic) MeOH, cat. HCl, r.t., 30 min.

With the three caged imidazopyrazinone compounds in hand we moved on to evaluating their response and selectivity towards Cu(II) in the presence of their cognate luciferases (Figure S2). We assessed pic-DTZ and pic-CTZ400a with recombinant Nanoluciferase (rNluc) and pic-CTZ with *Gaussia* luciferase.

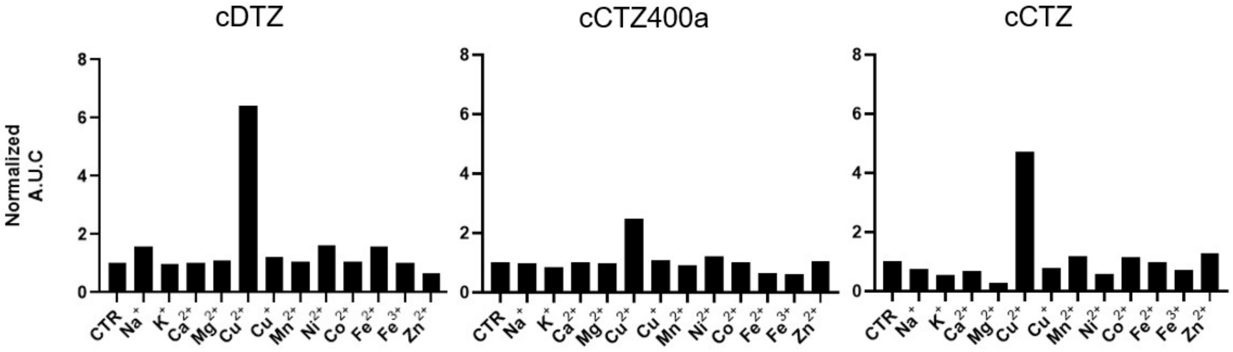

Figure S3. Calculated area under the curve of luminescence measured over 20 minutes with 5 μM caged probes in the presence of biologically-relevant d-block (5 μM) and s-block (1 mM) metals. For pic-DTZ and pic-CTZ400a, 120 nM rNluc was used. For pic-CTZ, 425 nM recombinant *Gaussia* luciferase was used.

pic-DTZ in the presence of rNluc exhibits a stronger intensity in signal (recorded over 20 minutes) relative to either pic-CTZ400a and pic-CTZ. Additionally, although pic-CTZ400a did show Cu(II)-selective light output, the signal-to-background ratio is much lower compared to either pic-DTZ or pic-CTZ. It should also be noted that access to native coelenterazine is more synthetically challenging relative to the other two imidazopyrazinones tested. Indeed the yield for the caged CTZ was so low we were unable to get a reasonable carbon NMR spectra and even the proton NMR displayed low signal to noise. For these reasons, we chose to move forward with pic-DTZ as the probe of choice. AS

Additional Supplementary Figures (Figures S4 - S12)

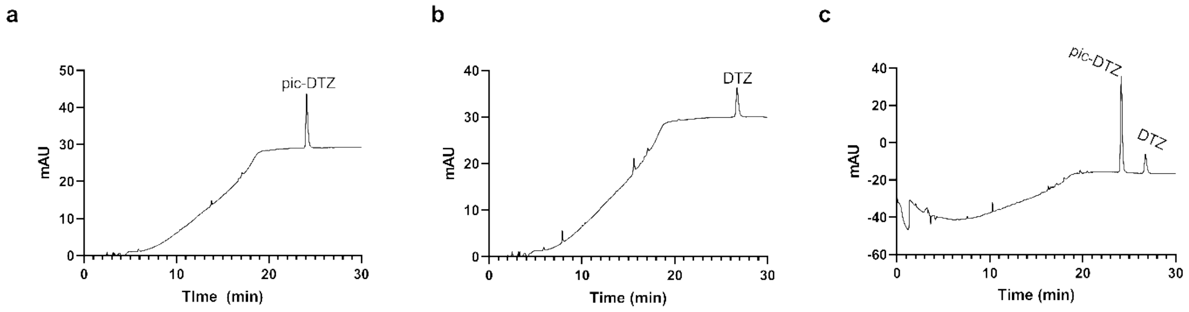

Figure S4. LCMS chromatograms of (a) pic-DTZ (50 μM) alone (b) pic-DTZ (50 μM) immediately after addition of 1 mM Cu(II) and (c) pic-DTZ (50 μM) 4 hours after addition of 5 μM Cu(II), monitored at 250 nm. All LCMS samples were prepared methanol. A gradient was run from 90:10 H_2O:MeCN + 0.1% FA to 10:90 H_2O:MeCN + 0.1% FA over 15 minutes, then held at 10:90 H_2O:MeCN + 0.1% FA for 15 minutes. Peak labeled "pic-DTZ" corresponds to m/z of 589.2 corresponding to $[M+H]^+$ and peak labeled "DTZ" corresponds to m/z of 376.2 corresponding to a salt form of DTZ.

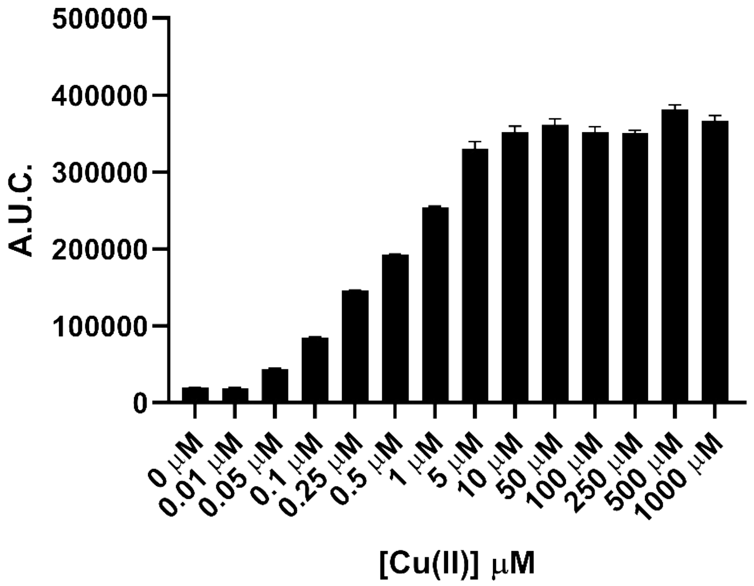

Figure S5. Dose-dependence of pic-DTZ (1 μM) with varying concentrations of Cu(II) ($CuSO_4$) in the presence of 120 nM rNluc.

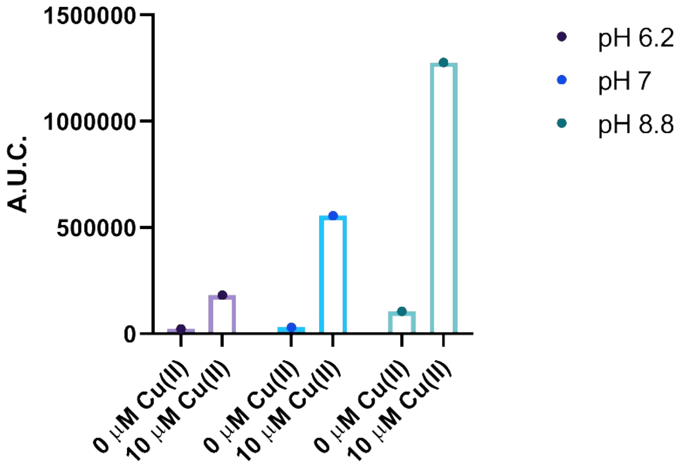

Figure S6. Calculated area under the curve for luminescence over 20 minutes of 1 μM pic-DTZ in the presence of 120 nM rNluc at various pHs in DPBS.

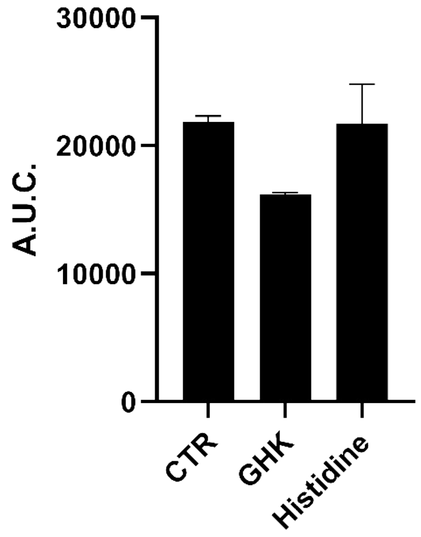

Figure S7. Calculated area under the curve over 20 minutes of 1 µM pic-DTZ in the presence of GHK and histidine (10 µM) with 120 nM rNluc. Error bars denote SEM, n = 3.

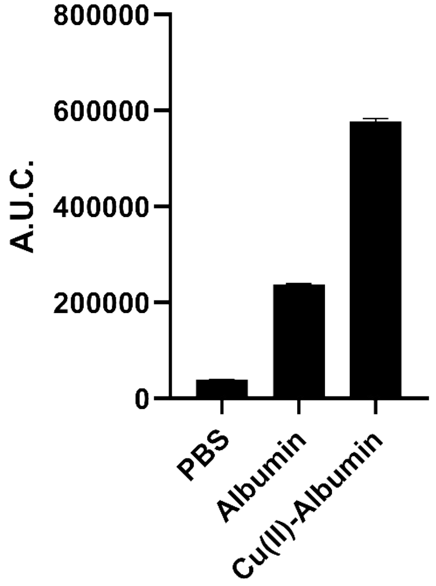

Figure S8. Calculated area under the curve over 20 minutes of 1 µM pic-DTZ in the presence of albumin (100 µM) or copper loaded albumin (1:1 molar ratio, 100 µM) with 120 nM rNluc. Error bars denote SEM, n = 3.

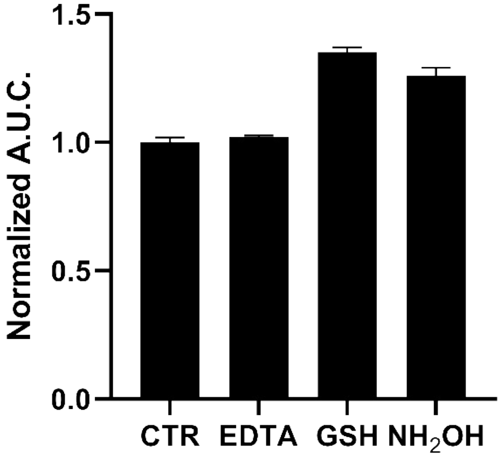

Figure S9. Calculated area under the curve over 20 minutes of 1 μM pic-DTZ in the presence of EDTA (1 mM), GSH (200 μM), or NH_2OH (200 μM) with 120 nM rNluc. Error bars denote SEM, n = 3.

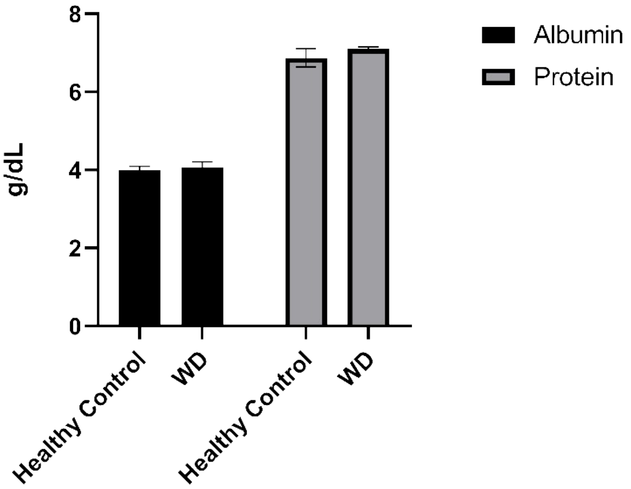

Figure S10. Clinically determined albumin and overall protein levels of healthy control and Wilson's diseased (WD) cohorts. Error bars denote SEM, n=3.

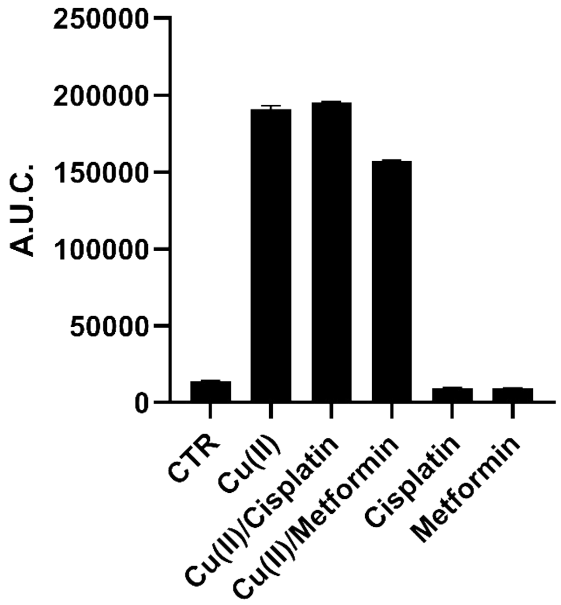

Figure S11. Calculated area under the curve over 20 minutes of 1 μM pic-DTZ in the presence of Cu(II) (10 μM) or cisplatin and metformin (30 μM and 5 mM respectively) with or without 10 μM Cu(II) and 125 nM rNluc. Error bars denote SEM, n = 3.

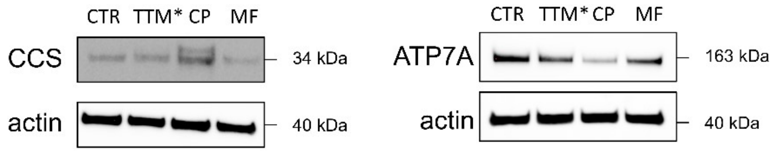

Figure S12. Western blot analysis of the copper chaperone for superoxide dismutase (CCS), and the copper transporter ATP7A, from cell lysates of secNluc MDA-MB-231 cells treated with 30 μM cisplatin (CP) or 5 mM metformin (MF) compared to untreated control (CTR). *TTM is included as a copper chelation control for the western blot experiments, but is not assessed with pic-DTZ as we observed light inhibition with native DTZ in the presence of TTM.

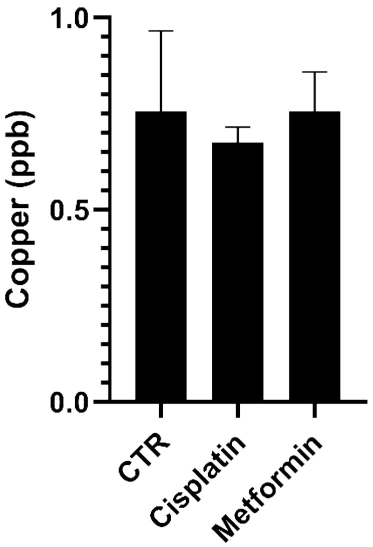

Figure S13. ICP-MS quantification of copper from cell media removed from secNluc MDA-MB-231 cells after treatment for 18 hours by cisplatin (30 μM) or metformin (5 mM) relative to untreated control (CTR). Error bars denote SEM, n=3.

Supplementary Materials and Methods

Synthesis of Heterocyclic Ester Cages

Thiophene ester, 7a. Phenol **6** (200 mg, 0.840 mg, 1 equiv.) and thiophene-2-carbonyl chloride (148 mg, 1.01 mmol, 1.2 equiv.) were added to a flame-dried round-bottom flask purged with N_2 and then dissolved in anhydrous pyridine/DMF (1:1, 2 mL) and allowed to stir at room temperature for three hours. The reaction was then quenched in water and extracted into ethyl acetate (3 X 10 mL). The combined ethyl acetate extract was then washed with brine, dried over Na_2SO_4 and concentrated under reduced pressure. The crude product was purified by silica gel column chromatography (70/30 hexanes/EtOAc) to obtain the desired product **7b** (224 mg, 77%) as a yellow oil. ^{1}H NMR (400 MHz, CDCl3) δ 7.98-8.00 (dd, 1H), 7.64-7.66 (dd, 1H), 7.37-7.39 (d, 2H), 7.16-7.20 (m, 3H), 4.72 (s, 2H), 0.97 (s, 9H), 0.13 (s, 6H). 13C NMR (100 MHz, $CDCl_3$) δ 160.82, 149.51, 139.32, 134.75, 133.56, 133.06, 128.12, 127.80, 127.16, 121.48, 115.16, 64.87, 64.55, 26.07, 18.52, -5.12. Low-resolution mass spectrometry (LRMS) (*m/z*): $[M+H]^+$ calculated for: $C_{18}H_{25}O_3SSi$, 349.1; found $[M+H]^+$ 349.1.

Furan ester, 7b. Phenol **6** (200 mg, 0.840 mg, 1 equiv.) and furan-2-carbonyl chloride (132 mg, 1.01 mmol, 1.2 equiv.) were added to a flame-dried round-bottom flask purged with N_2 and then dissolved in anhydrous pyridine/DMF (1:1, 2 mL) and allowed to stir at room temperature for three hours. The reaction was then quenched in water and extracted into ethyl acetate (3 X 10 mL). The combined

ethyl acetate extract was then washed with brine, dried over Na_2SO_4, and concentrated under reduced pressure. The crude product was purified by silica gel column chromatography (70/30 hexanes/EtOAc) to obtain the desired product **7b** (230 mg, 83%) as a white solid. ^{1}H NMR (400 MHz, CDCl3) δ 7.66 (s, 1H), 7.36-7.38 (m, 3H), 7.16-7.18 (d, 2H), 4.75 (s, 2H), 0.95 (s, 9H), 0.11 (s, 6H). 13C NMR (100 MHz, CDCl3) δ 159.77, 149.11, 147.22, 144.57, 139.44, 127.19, 121.85, 119.92, 112.30, 64.52, 26.54, 17.19, -5.13. Low-resolution mass spectrometry (LRMS) (*m/z*): $[M+H]^+$ calculated for $C_{18}H_{25}O_4Si$, 331.15; found $[M+H]^+$ 331.1.

Synthesis of pic-CTZ400a

3-benzyl-5-bromopyrazin-2-amine, 3b. Zinc mesh 20-30 (1.18 g, 18 mmol, 3.5 equiv.), zinc dust (1.18 g, 18 mmol, 3.5 equiv.), and iodine (70 mg, 0.9 mmol, 5 mol%) were added to a flame-dried round-bottom flask purged with N_2. Benzyl bromide (967 mg, 5.65 mmol, 1.1 equiv.) in N,N-DMA (8 mL) was then added to the round-bottom flask and the reaction was heated to 80 ºC and allowed to stir for three hours. A suspension of 3,5-dibromo-2-aminopyrazine (1.3 g, 5.14 mmol, 1 equiv.) and $PdCl_2(PPh_3)_2$ (180 mg, 0.257 mmol, 5 mol%) in N,N-DMA (8 mL) was added to the reaction mixture. The reaction was allowed to stir for 72 hours. The reaction was quenched in water (100 mL) and extracted with ethyl acetate (3 X 25 mL). The combined ethyl acetate extract was washed with brine, dried over Na_2SO_4, and concentrated under reduced pressure. The crude product was then purified by silica gel column chromatography (60/40 hexanes/EtOAc) to afford **3b** (1.25 g, 92%) as a brown solid. ^{1}H NMR (400 MHz, $CDCl_3$) δ 8.02 (s, 1H), 7.26-7.36 (m, 5H), 4.40 (bs, 2H), 4.08 (s, 2H). Low-resolution mass spectrometry (LRMS) (*m/z*): $[M+H]^+$ calculated for $C_{11}H_{10}BrN_3$, 264.0; found $[M+H]^+$ 264.0.

3-benzyl-5-phenylpyrazin-2-amine, 4b. 3-benzyl-5-bromopyrazin-2-amine, **3b**, (339 mg, 1.283 mmol, 1 equiv.), phenylboronic acid (188 mg, 1.54 mmol, 1.2 equiv.), $Pd(PPh_3)_4$ (148 mg, 0.128 mmol, 0.1 equiv.), and K_2CO_3 (310 mg, 2.25 mmol, 1.75 equiv.) were all added to a round-bottom flask purged with N_2, then water (5 mL) and 1,4-dioxane (25 mL) were added. The reaction was heated to 80 ºC and was allowed to stir overnight. The reaction was then quenched in water (100 mL) and extracted into ethyl acetate (3 X 25 mL). The combined ethyl acetate extract was then washed with brine, dried over Na_2SO_4, and concentrated under reduced pressure. The crude product was then purified by silica gel column chromatography (70/30 pet. ether/hexanes) to afford **3c** (282 mg, 84%) as a brown solid.^{1}H NMR (400 MHz, DMSO) δ 8.43 (s, 1H), 7.90-7.92 (d, 2H), 7.27-7.43 (m, 7H), 7.20 (t, 1H), 6.40 (s, 2H), 4.09 (s, 2H). Low-resolution mass spectrometry (LRMS) (*m/z*): $[M+H]^+$ calculated for $C_{17}H_{16}N_3$, 262.1; found 262.2.

CTZ400a, 5b. 3-benzyl-5-phenylpyrazin-2-amine, **4b,** (217 mg, 0.830 mmol, 1 equiv.) and 1,1-diethoxy-3-phenylpropan-2-one, **10**, (277 mg, 1.246 mmol, 1.5 equiv.) were added to a N_2-purged round-bottom flask followed by ethanol (16 mL), water (2 mL), and hydrochloric acid (0.4 mL). The reaction was then refluxed overnight. The reaction was then concentrated under reduced pressure and purified on alumina gel column (gradient 0-10% MeOH in DCM) to afford **5b** (150 mg, 46%) of pure product as a bright yellow solid. ^{1}H NMR (400 MHz, DMSO) δ 7.16-7.92 (m, 16H), 4.34 (s, 2H), 4.09 (s, 2H). Low-resolution mass spectrometry (LRMS) (*m/z*): $[M+H]^+$ calculated for: $C_{26}H_{22}N_3O$, 392.18; found $[M+H]^+$ 392.1.

pic-CTZ400a. CTZ400a, **5b**, (86 mg, 0.219 mmol, 1 equiv.), 4-(bromomethyl)phenyl picolinate, **9**, (128 mg, 0.439 mmol, 2 equiv.), Cs_2CO_3 (30 mg, 0.087 mmol, 0.4 equiv.), and KI (40 mg, 0.241 mmol, 1.1 equiv.) were added to a flame-dried round-bottom flask that was then purged with N_2. The reagents were then dissolved in anhydrous MeCN (2 mL) and the reaction was allowed to stir overnight. The following morning the reaction mixture was quenched in 20 mL of water and extracted in DCM (3 x 10 mL). The combined DCM extract was then washed with brine, dried over Na_2SO_4, and concentrated under reduced pressure. The crude product was then purified by reverse-phase HPLC (gradient, 70% H_2O/30% MeCN to 100% MeCN over 50 minutes on an Agilent C3 column) and dried under reduced pressure to obtain **pic-CTZ400a** (31 mg, 24% yield) as a brown-red solid. ^{1}H NMR (800 MHz, CD_2Cl_2) δ 8.80-8.82 (d, 1H), 8.22-8.24 (d, 1H), 7.87-7.97 (m, 4H), 7.54-7.58 (m, 3H), 7.17-7.47 (m, 15H), 5.03 (s, 2H), 4.55 (s, 2H), 4.13 (s, 2H). ^{13}C NMR (200 MHz, CD_2Cl_2) δ 152.54, 151.48, 139.40, 138.31, 138.17, 137.14, 136.99, 133.80, 133.62, 132.53, 130.82, 130.34, 129.99, 129.55, 128.97, 128.79, 128.70, 128.43, 128.29, 128.20, 127.52, 126.82, 126.35, 126.32, 126.11, 125.72, 124.98, 122.11, 109.32, 76.57, 39.32, 33.34, 29.68. Low-resolution mass spectrometry (LRMS) (*m/z*): $[M+H]^+$ calculated for: $C_{39}H_{31}N_4O_3$, 603.24; found $[M+H]^+$ 603.2.

Synthesis of pic-CTZ

pic-CTZ Coelenterazine (Goldbio), (25 mg, 0.06 mmol, 1 equiv.), 4-(bromomethyl)phenyl picolinate, **9**, (20 mg, 0.07 mmol, 1.1 equiv.), Cs_2CO_3 (8 mg, 0.02 mmol, 0.4 equiv.), and KI (10 mg, 0.07 mmol, 1.1 equiv.) were added to a flame dried round-bottom flask that was then purged with nitrogen. The reagents were then dissolved in anhydrous acetonitrile (0.5 mL) and the reaction was allowed to stir overnight. The following morning the reaction mixture was quenched in 20 mL of water and extracted in DCM (3 x 10 mL). The combined DCM extract was then washed with brine, dried over Na_2SO_4, and concentrated under reduced pressure. The crude product was then purified by reverse-phase HPLC

(gradient, 70% H_2O/30%MeCN over 50 minutes 100% MeCN on a T3 Atlantis column) and dried under reduced pressure to obtain **pic-CTZ** (0.5 mg, 1.3% yield) as a brown-red solid. Low-resolution mass spectrometry (LRMS) (*m/z*): $[M+H]^+$ calculated for: $C_{39}H_{30}N_4O_5$, 635.23; found $[M+H]^+$ 635.3.

Evaluation of pH responsiveness of pic-DTZ

DPBS solutions were prepared from a stock commercial solution to desired pH using either 6 M HCl or NaOH and then 125 nM rNluc solutions were made from a stock solution of rNluc (Promega) and these were plated in a 96-well white, opaque plate. To the wells were added Cu(II) to a final concentration of 10 μM when appropriate. Lastly, 1 μL of a 100 μM pic-DTZ (in ethanol) solution was added to all wells and the luminescence was measured immediately as previously described.

ICP-MS analysis of copper in cell media

Cells were plated at 100,000 cells per well in a 12-well clear, flat-bottom plate. Eight hours later, the media was removed and the cells were washed with pre-warmed (37 ºC) DPBS and 100 μL of OptiMem (Gibco) was added to each well. A 3 mM solution of cisplatin and 500 mM solution of metformin were prepared in water. 10 μL of these solutions were added to the wells for final concentrations of 30 μM cisplatin and 5 mM metformin. At the 18-hour time point cell media was removed and was digested in concentrated acid overnight. The following morning the solutions were diluted in 2% (v/v) aqueous nitric acid. Metal analysis was performed at the Northwestern University Quantitative Bio-element Imaging Center generously supported by NASA Ames Research Center NNA06CB93G.

Cell Stimulations and Western Blot Analysis

secNluc MDA-MB-231 cells were plated in a 6 well plate at 200,000 cells per well. 6 hours after plating the media was removed, the cells were washed with prewarmed (37°C) DPBS, and fresh OptiMem was added. TTM, Cis-platin, and metformin solutions were prepared in water and added to wells at a final concentration of 30 μM, 30 μM, and 5 mM, respectively. At the 18-hour time point cells were lysed in RIPA buffer (150 mM NaCl, 1% NP-40, 0.5% sodium deoxycholate, 0.1% SDS, 50 mM Tris pH 7.4) with EDTA free protease inhibitor (ThermoFisher) and phosphatase inhibitor (Sigma). Lysates were put one ice for 15 minutes before being vortexed and centrifuged at 15000 x G at 4°C. Protein was quantified after freezing the lysates by BCA assay (Invitrogen). For Ctr1, ATP7A, and CCS, samples were made using 10 μg of protein with PBS (Gibco), sample buffer (Invitrogen), and 2-mercaptoethanol (BioRad). Samples were loaded into a 4-12% bis-tris 10 well gel (Invitrogen) and run for one hour at 100V and then was transferred on to a PVDF membrane using a Trans-Blot Turbo Transfer System (BioRad). The

membranes were blocked for one hour in 5% milk in TBST buffer. Membranes were then incubated overnight at 4°C with primary antibodies and washed three times the following day with TBST buffer before incubating secondary antibodies for one hour. Membranes were imaged on a Chemidoc MP Imager (BioRad). Primary antibodies used were Anti-Ctr1 (1:2,000 Cell Signaling Technologies), Anti-ATP7A (1:2,000 Santa Cruz Biotechnology), Anti-CCS (1:1,000 Santa Cruz Biotechnology), and Anti-Actin (Ms IgG and Rb IgG, 1:5000 or 1,10,000 respectively, Santa Cruz Biotechnology and Cell Signaling Technologies respectively). Secondary antibodies used were anti-rabbit IgG HRP-conjugated antibody (1:2,000 Cell Signaling Technologies) for Ctr1, anti-mouse IgG HRP-conjugated antibody (1:2,000 Cell Signaling Technologies) for ATP7A and CCS, anti-rabbit IgG Alexafluor 800 (1:10,000 Invitrogen) for actin and anti-mouse IgG Alexafluor 800 (1:5,000 Invitrogen) for actin.

NMR Spectra

3,5-dibromo-2-aminopyrazine, 2.

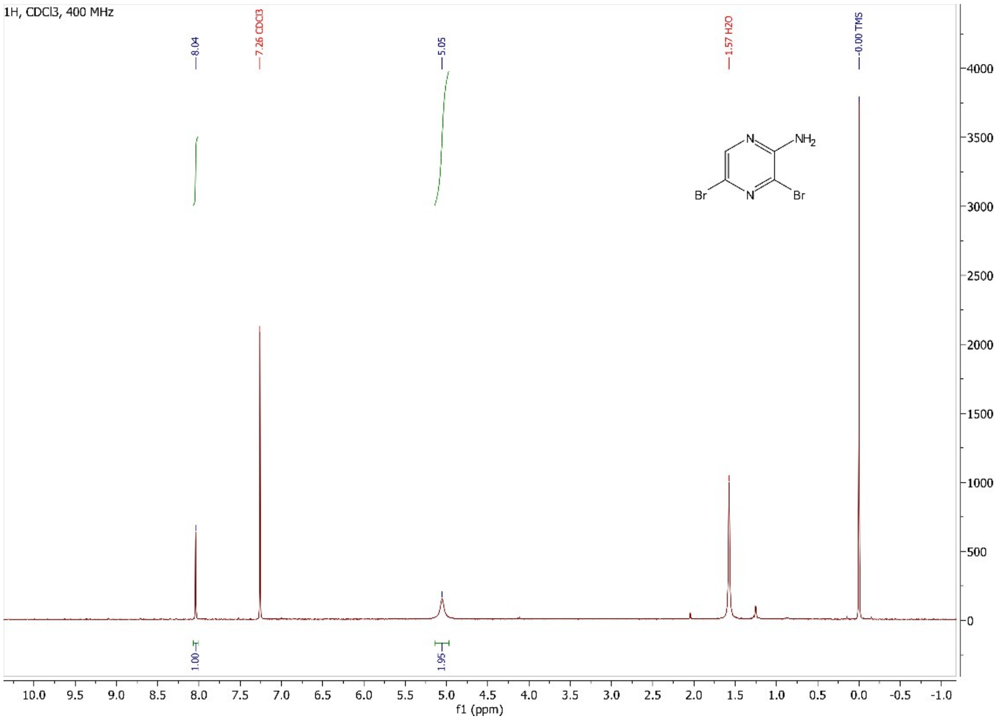

3,5-diphenyl-2-aminopyrazine, 3.

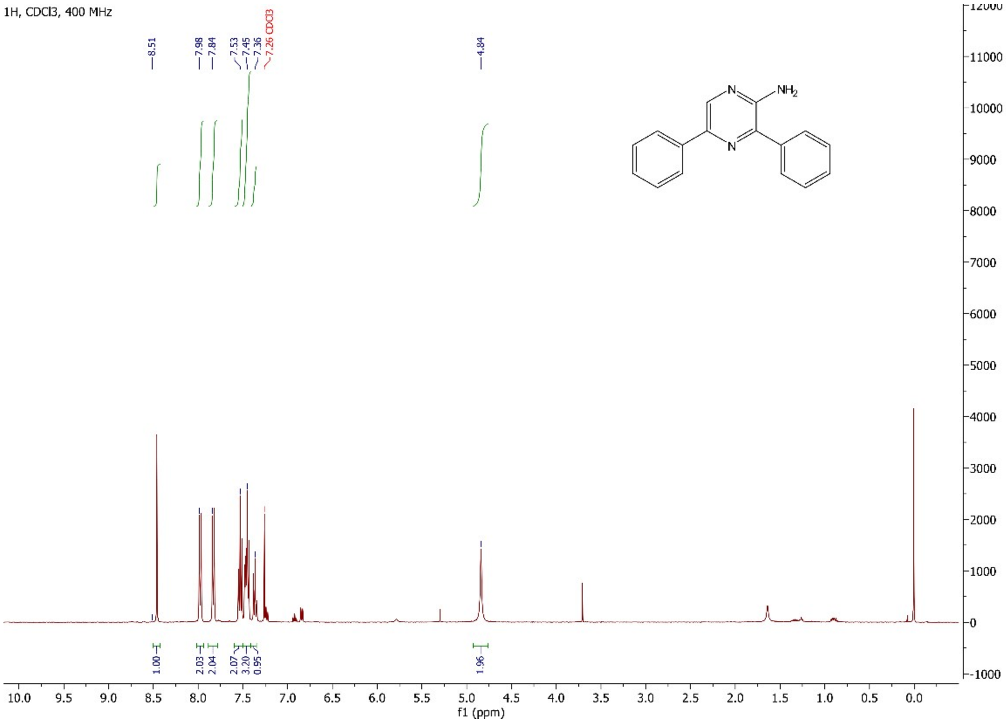

Diphenylterazine, 4.

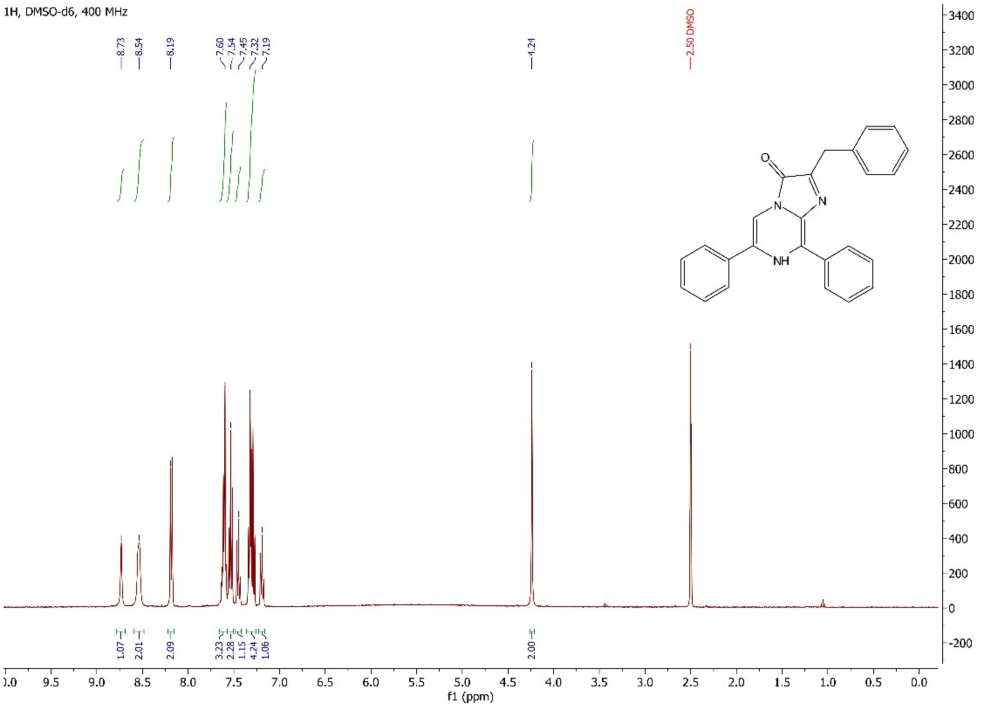

4-(((tert-butyldimethylsilyl)oxy)methyl)phenol, 6.

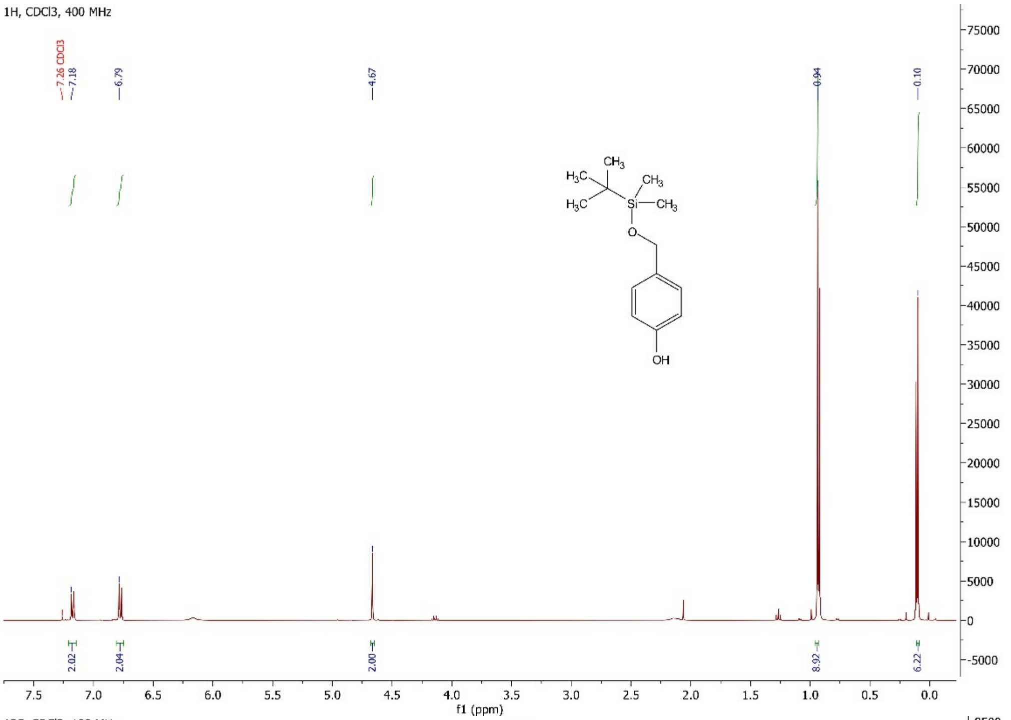

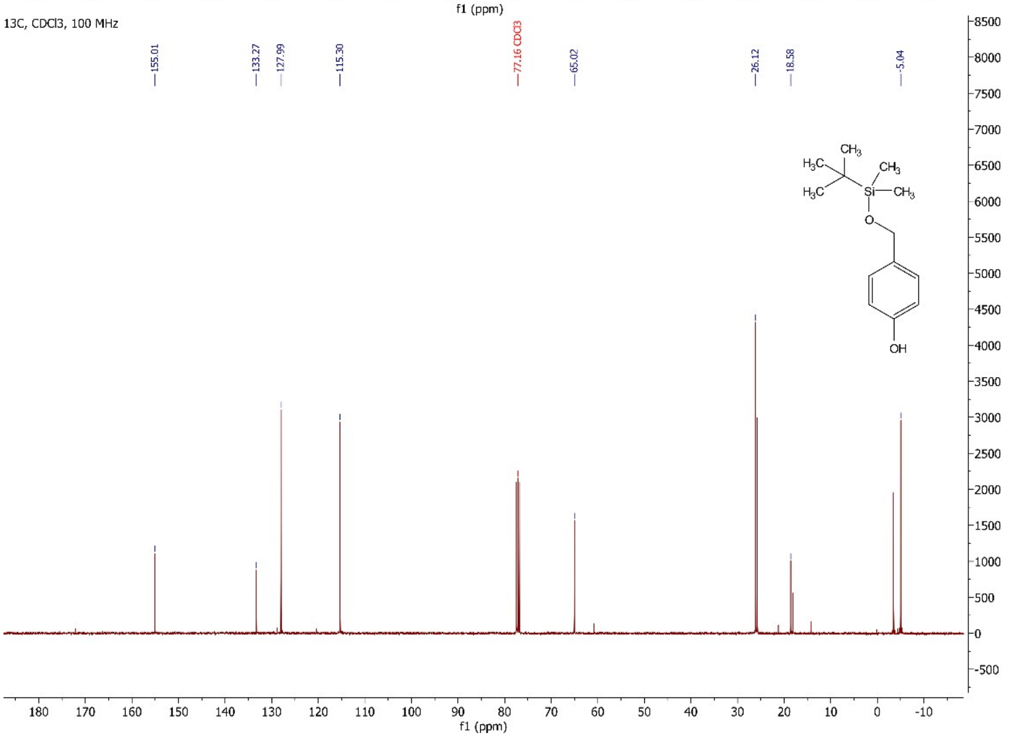

4-(((tert-butyldimethylsilyl)oxy)methyl)phenyl picolinate, 7.

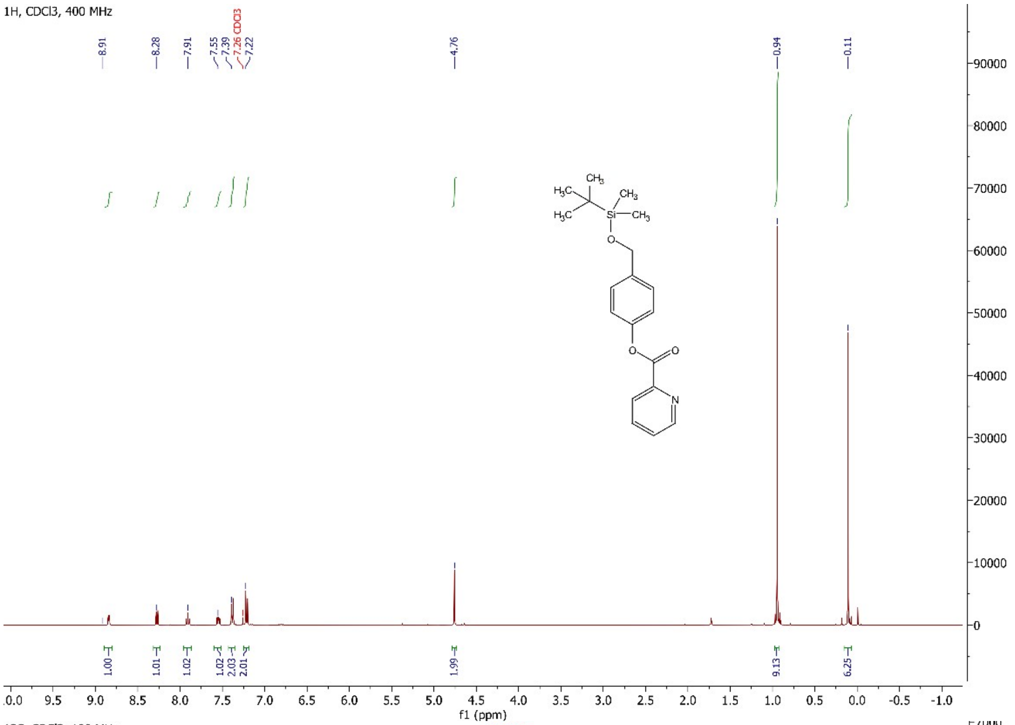

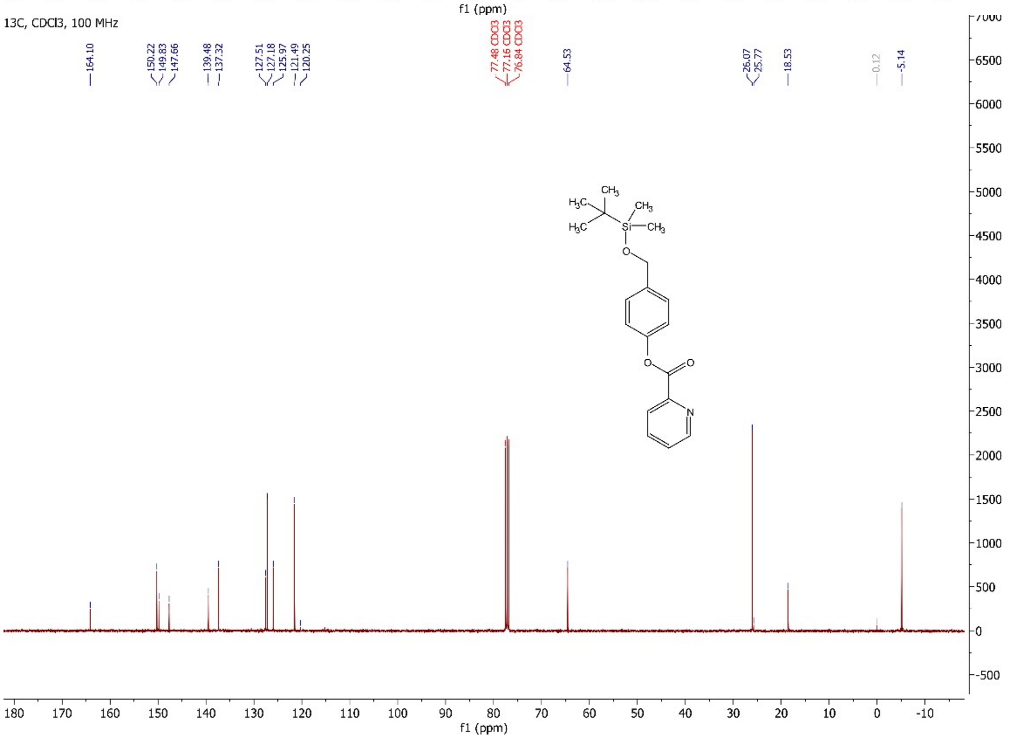

4-(hydroxymethyl)phenyl picolinate, 8

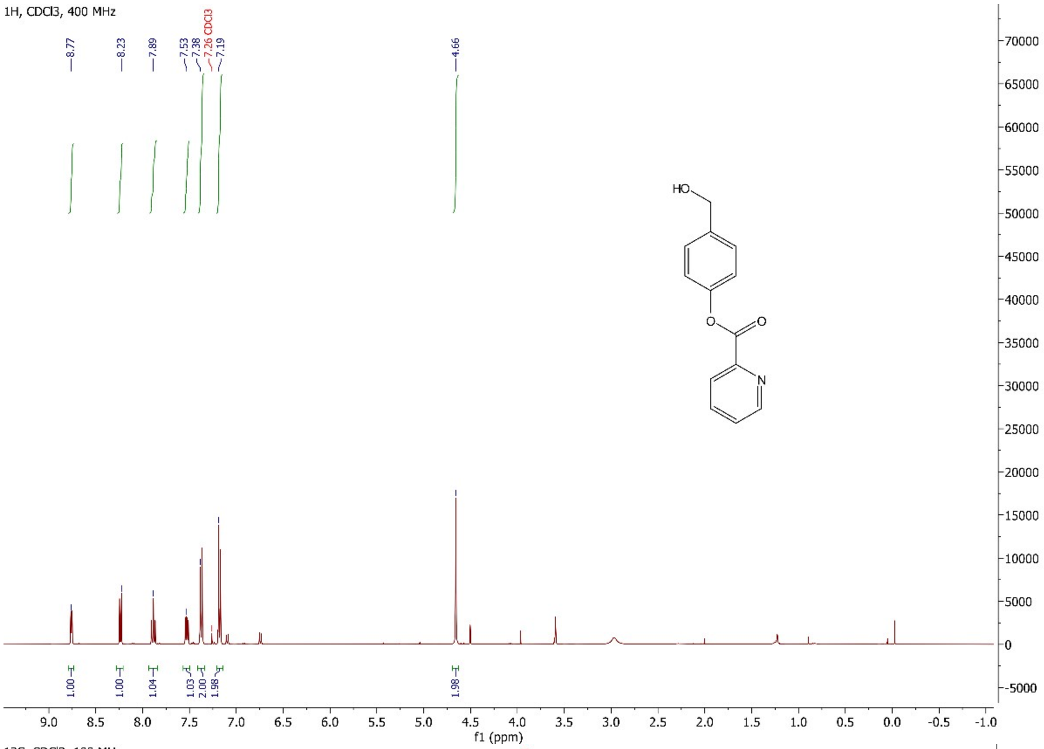

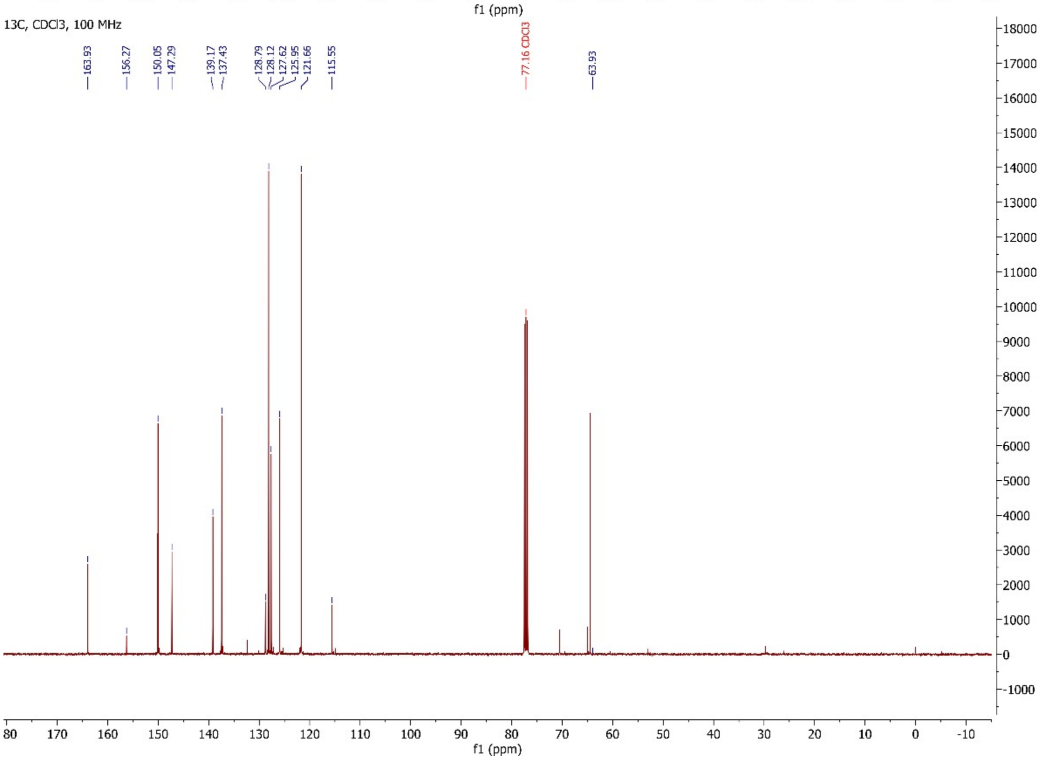

4-(bromomethyl)phenyl picolinate, 9.

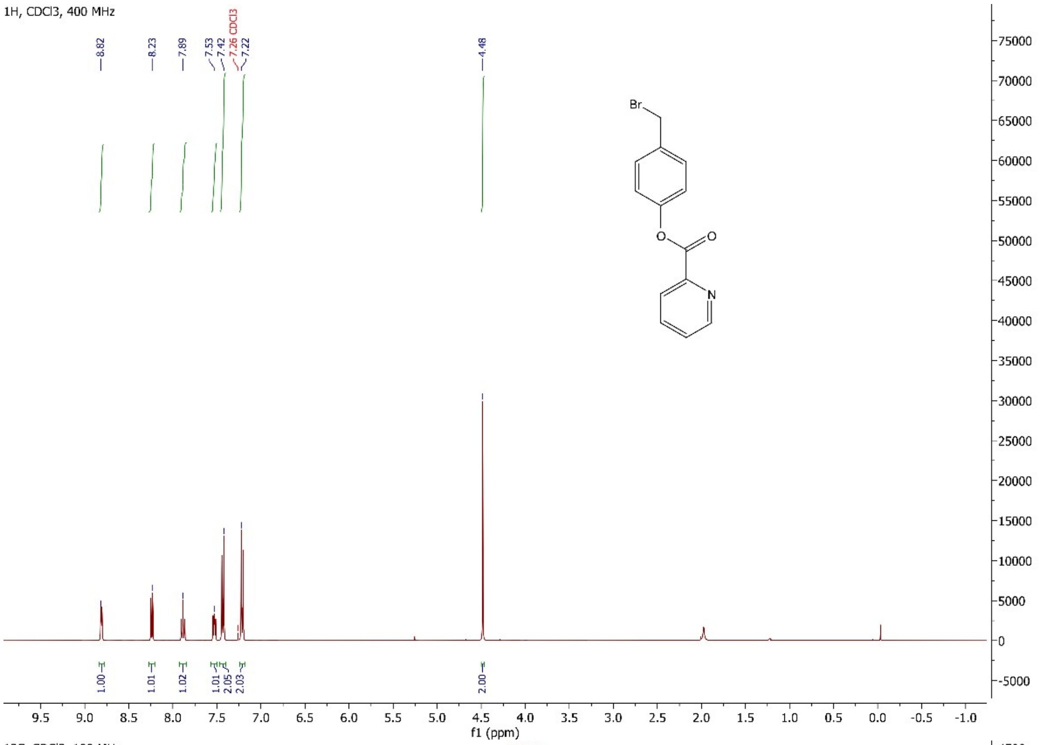
1H, CDCl3, 400 MHz
8.82
8.23
7.89
7.53
7.42
7.26 CDCl3
7.22
4.48
1.00
1.01
1.02
1.01
2.05
2.03
2.00
Br
O
O
N
f1 (ppm)

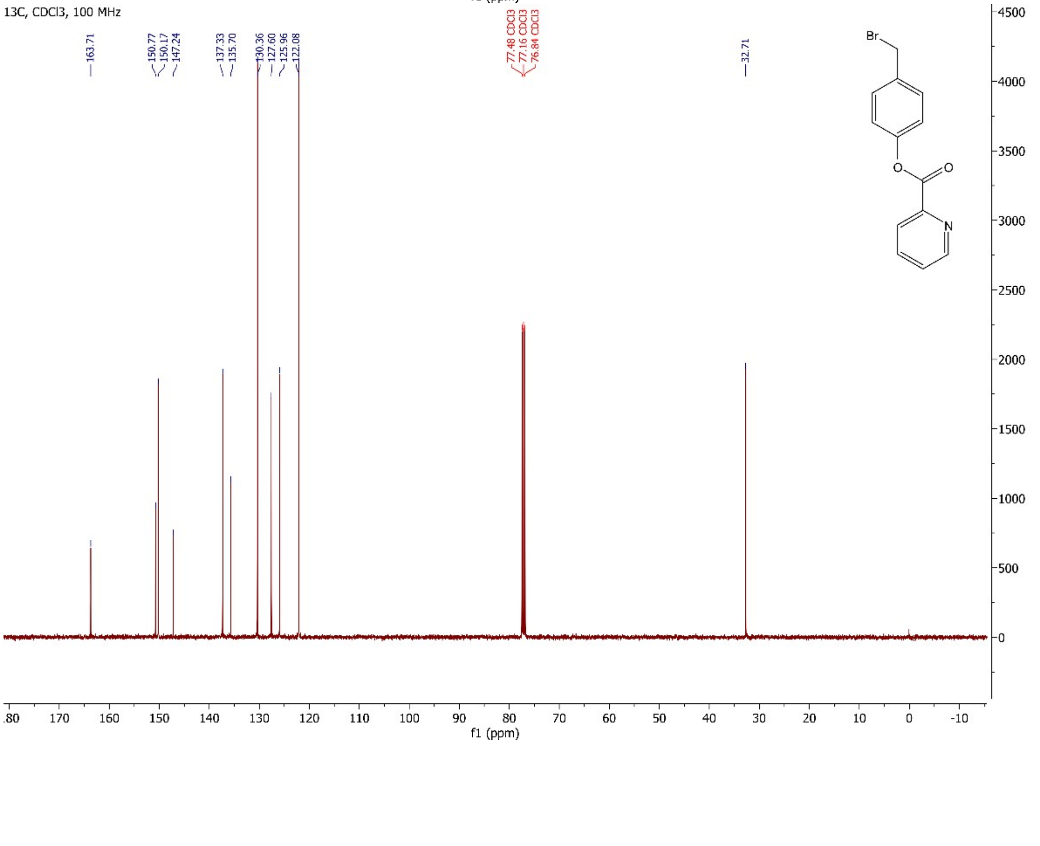
13C, CDCl3, 100 MHz
163.71
150.77
150.17
147.24
137.33
135.70
130.36
127.60
125.96
122.08
77.48 CDCl3
77.16 CDCl3
76.84 CDCl3
32.71
Br
O
O
N
f1 (ppm)

pic-DTZ

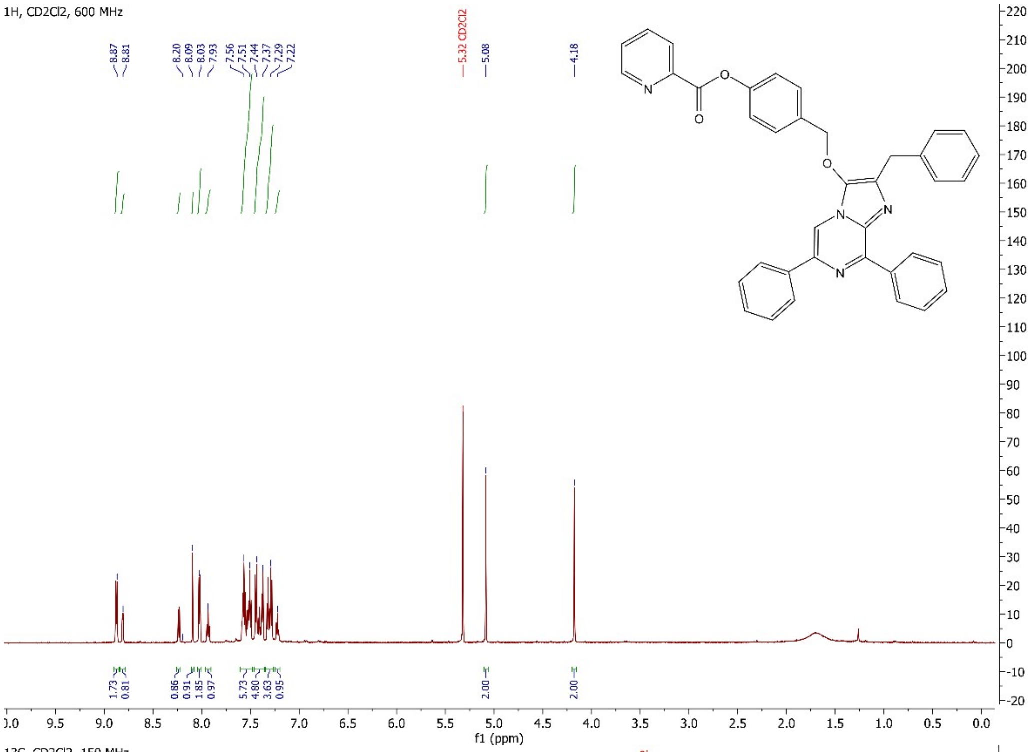

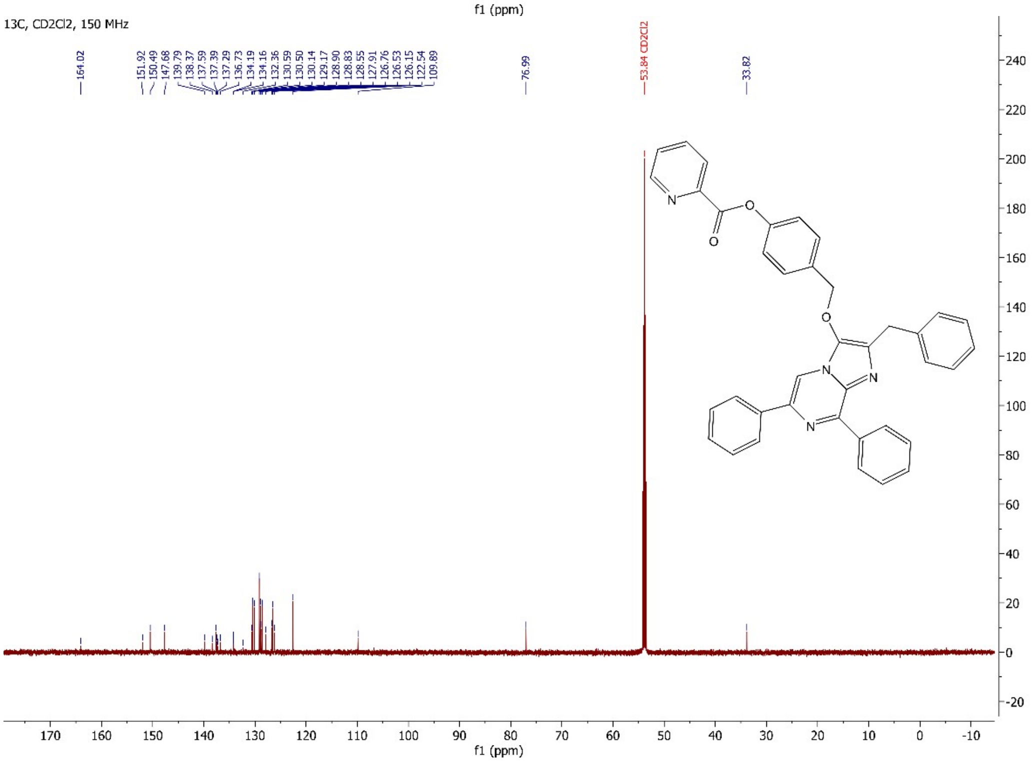

Thiophene Ester, 7a

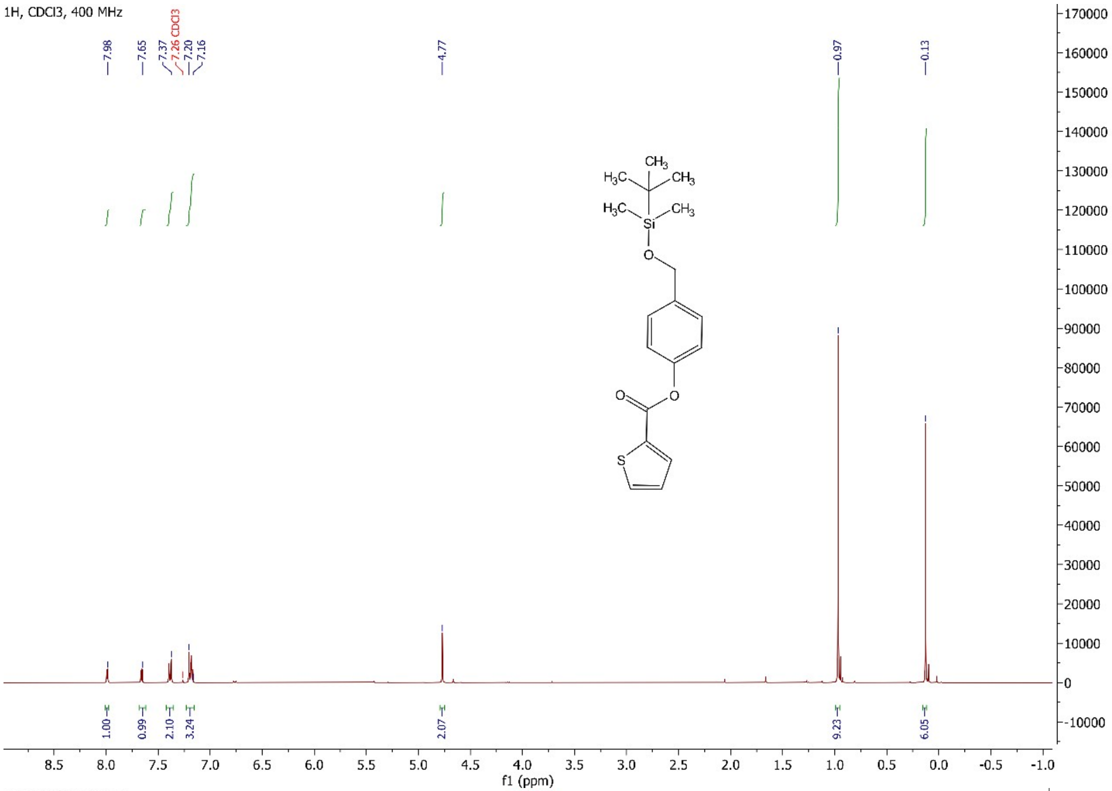

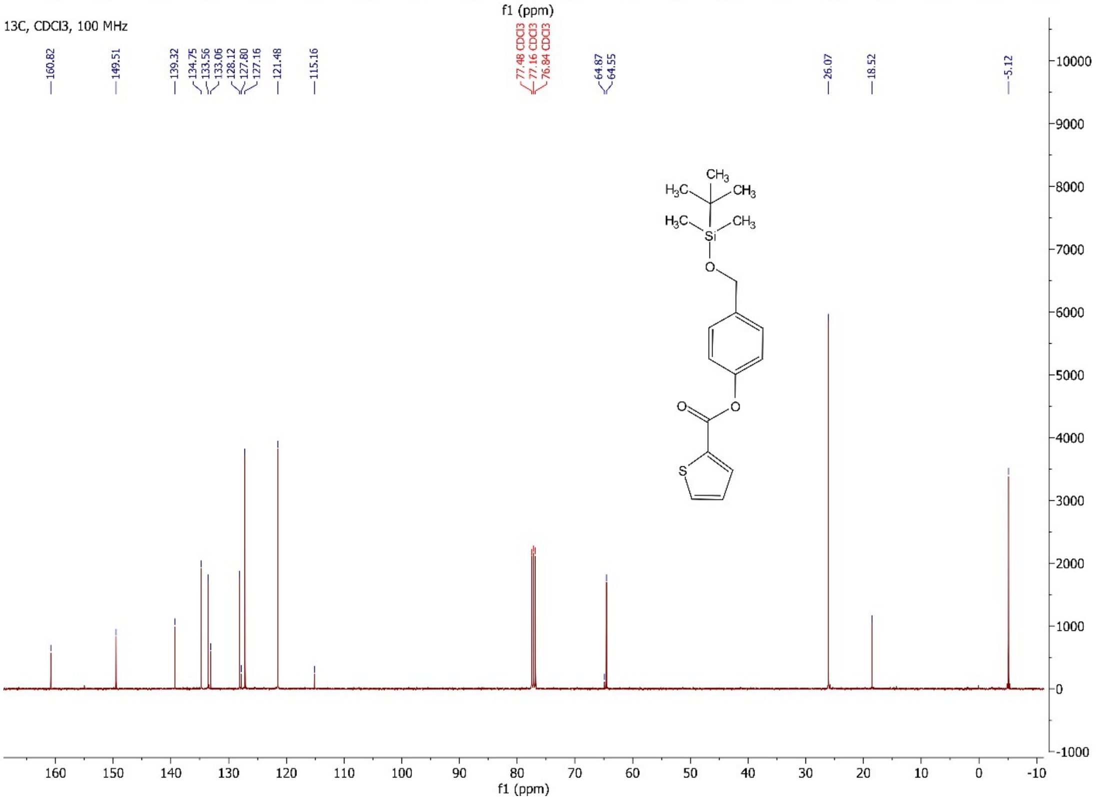

Furan ester, 7b

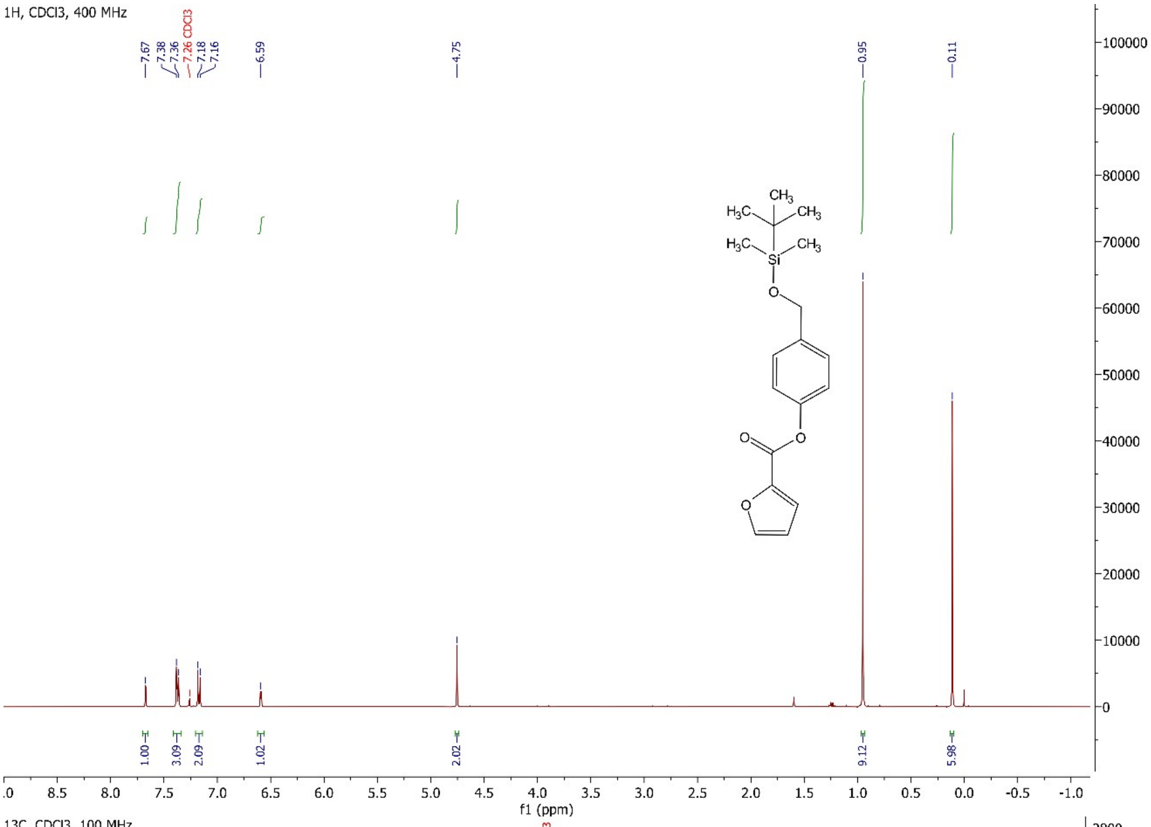

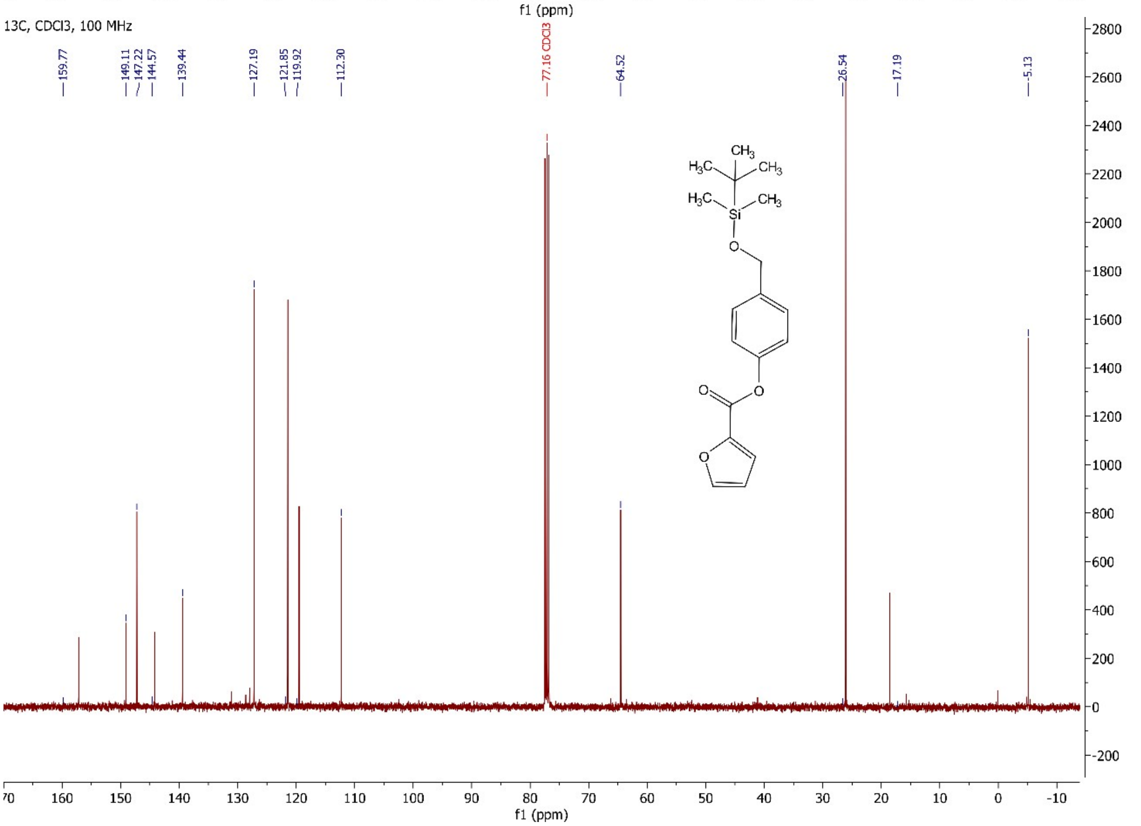

3-benzyl-5-bromopyrazin-2-amine, 3b

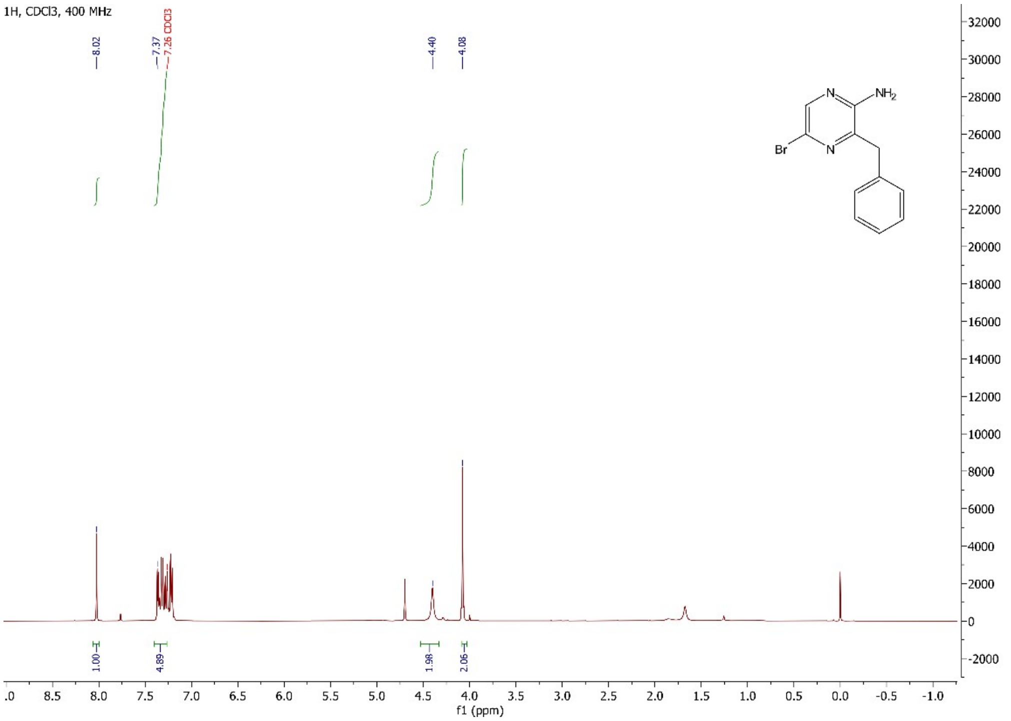

3-benzyl-5-phenylpyrazin-2-amine, 4b

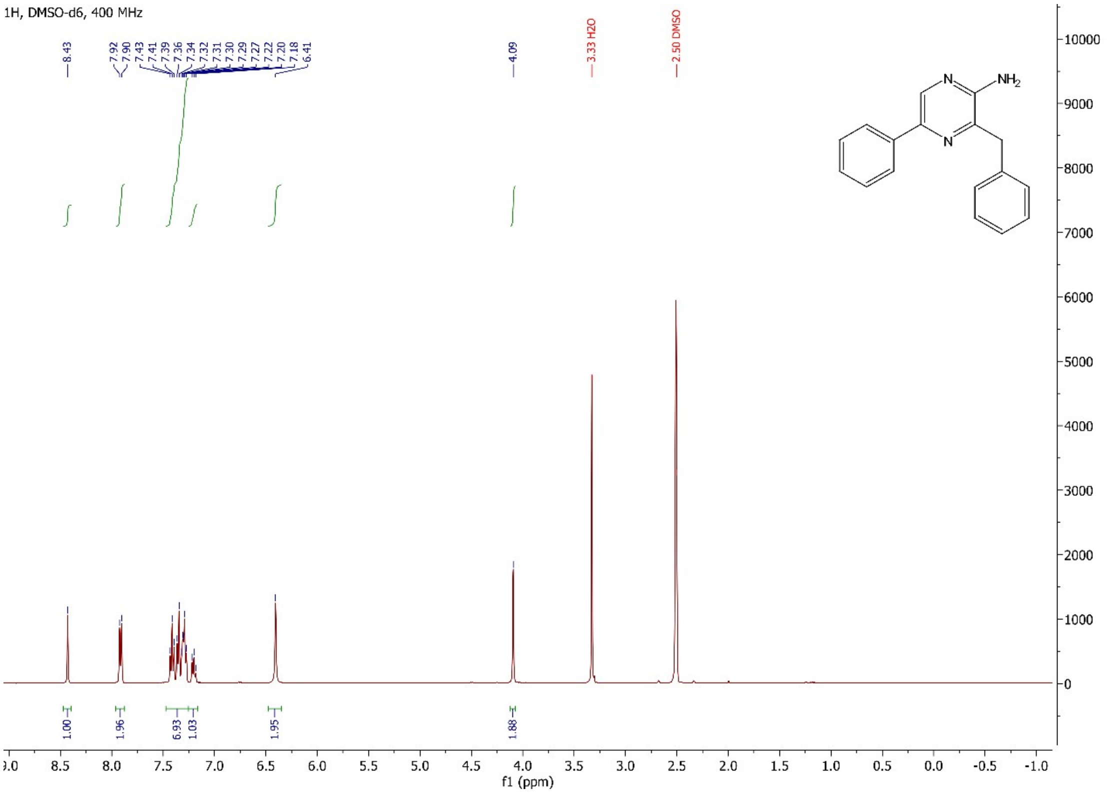

CTZ400a, 5b

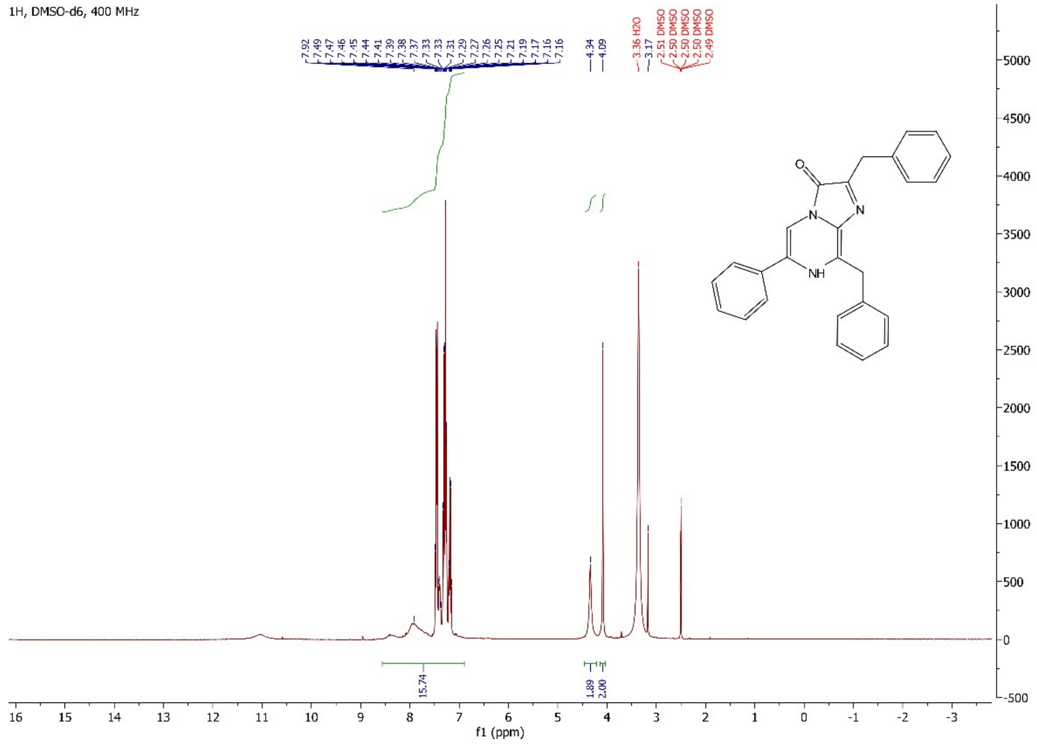
1H, DMSO-d6, 400 MHz
7.92
7.49
7.47
7.46
7.45
7.44
7.41
7.39
7.38
7.37
7.33
7.33
7.31
7.29
7.27
7.26
7.25
7.21
7.19
7.17
7.16
7.16
4.34
4.09
3.36 H2O
3.17
2.51 DMSO
2.50 DMSO
2.50 DMSO
2.50 DMSO
2.49 DMSO
O
N
N
NH
15.74
1.89
2.00
f1 (ppm)

pic-CTZ400a

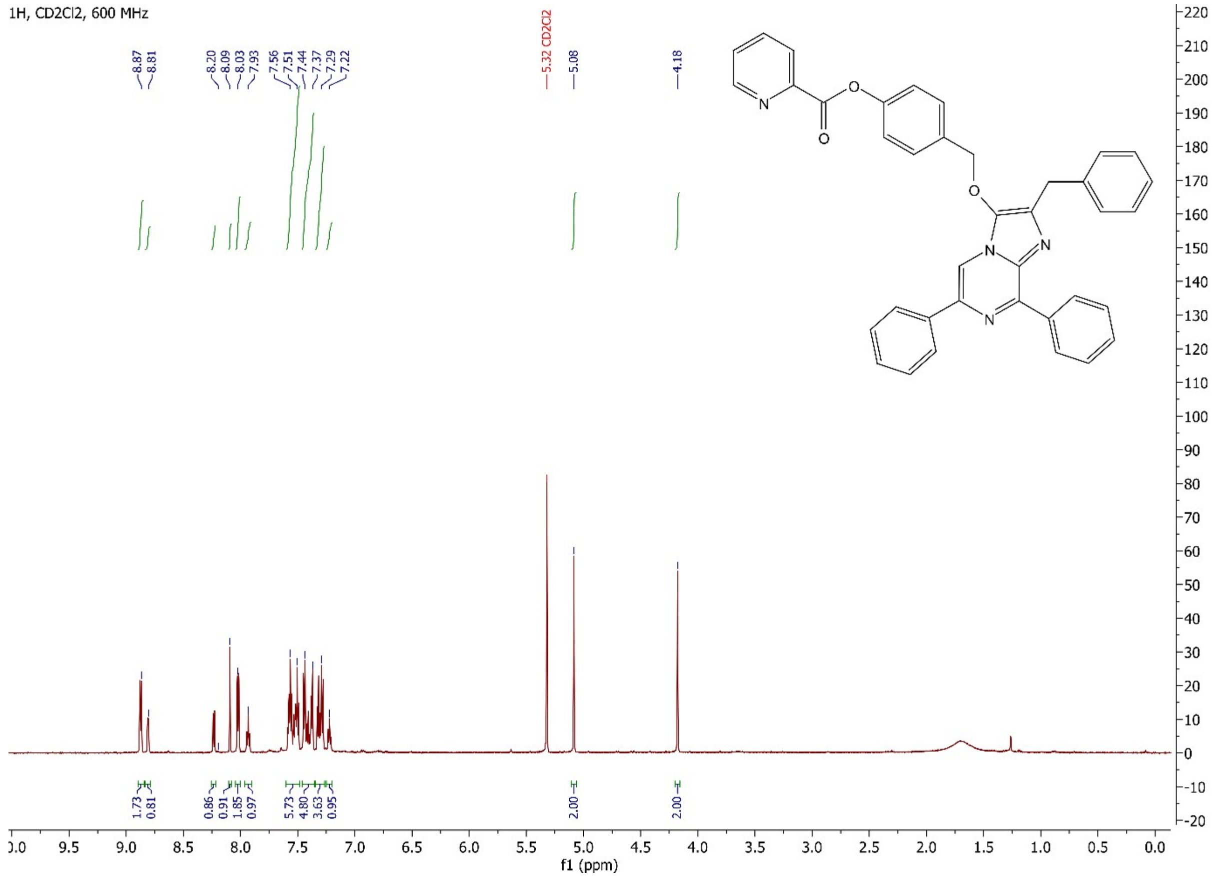
1H, CD2Cl2, 600 MHz
8.87
8.81
8.20
8.09
8.03
7.93
7.56
7.51
7.44
7.37
7.29
7.22
5.32 CD2Cl2
5.08
4.18
1.73
0.81
0.86
0.91
1.85
0.97
5.73
4.80
3.63
0.95
2.00
2.00
O
N
f1 (ppm)

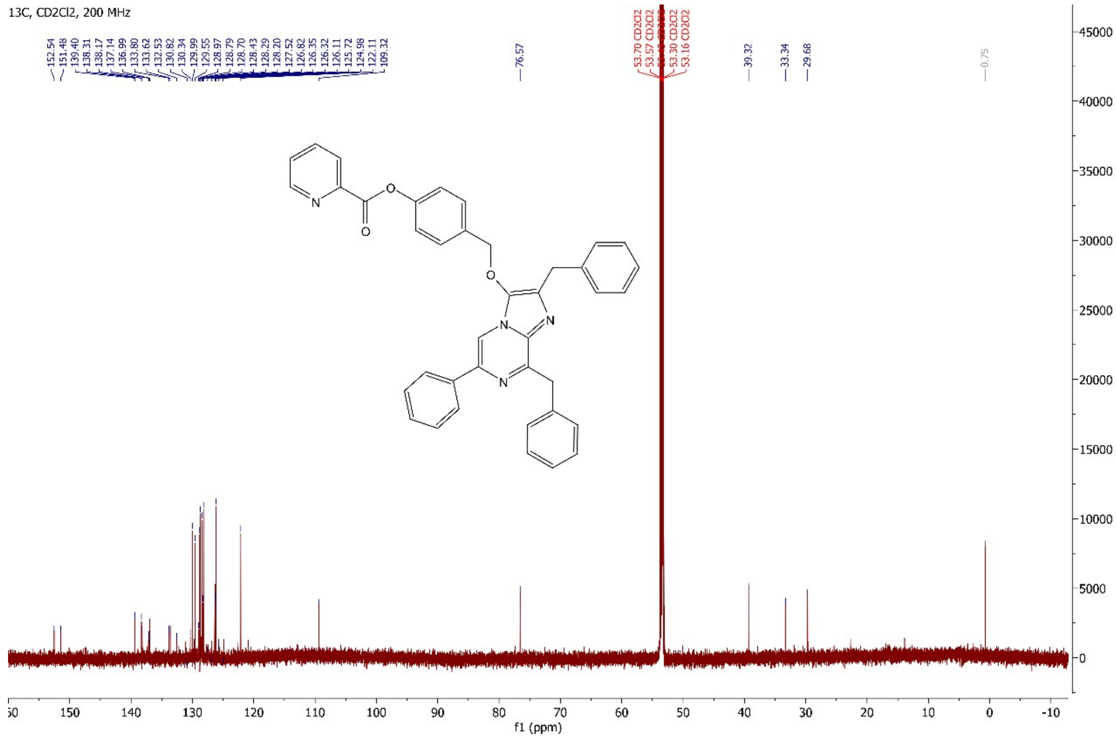
13C, CD2Cl2, 200 MHz
152.54
151.48
139.40
138.31
138.17
137.14
136.99
133.80
133.62
132.53
130.82
130.34
129.99
129.55
128.97
128.79
128.70
128.43
128.29
128.20
127.52
126.82
126.35
126.32
126.11
125.72
124.98
122.11
109.32
76.57
53.70 CD2Cl2
53.57 CD2Cl2
53.30 CD2Cl2
53.16 CD2Cl2
39.32
33.34
29.68
0.75
O
N
f1 (ppm)

cCTZ

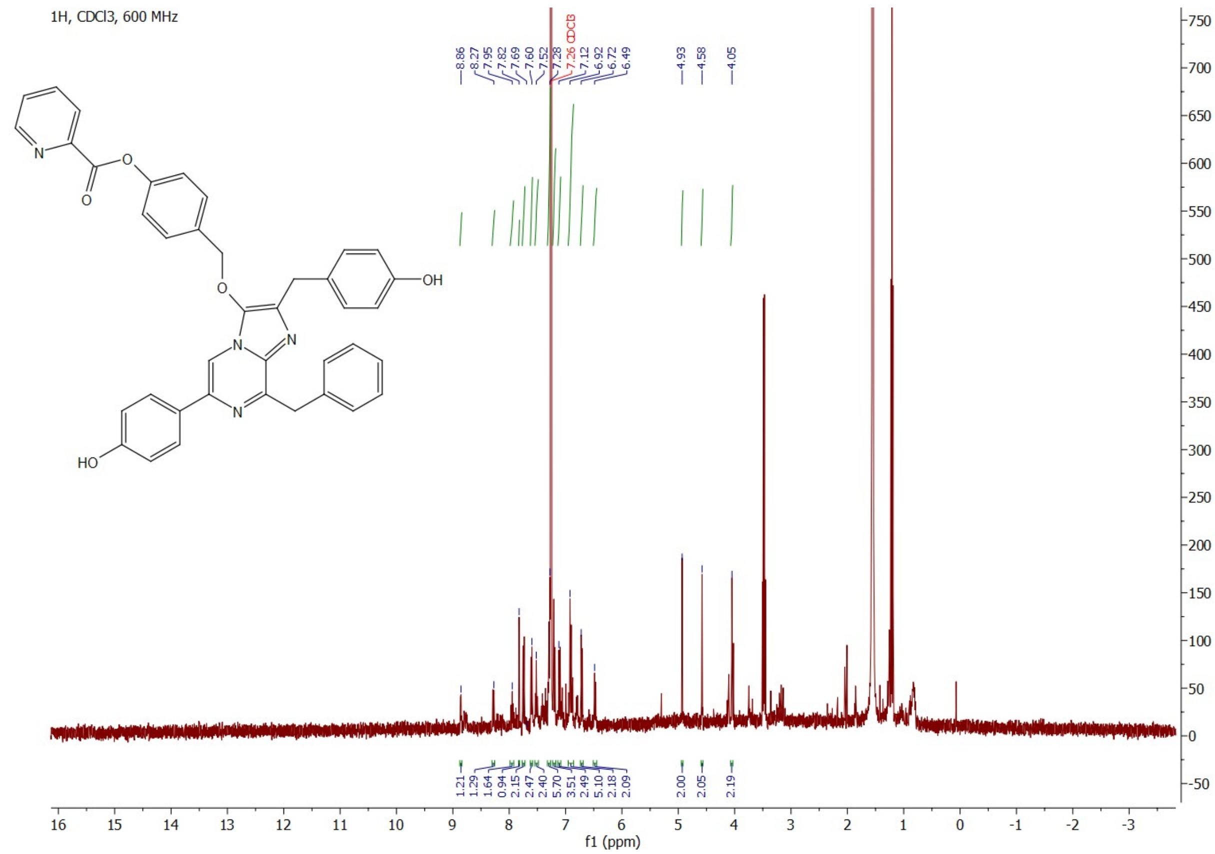

1H, CDCl3, 600 MHz
8.86
8.27
7.95
7.82
7.69
7.60
7.52
7.28
7.26 CDCl3
7.12
6.92
6.72
6.49
4.93
4.58
4.05
1.21
1.29
1.64
0.94
2.15
2.47
2.40
5.70
3.51
2.49
5.10
2.18
2.09
2.00
2.05
2.19
f1 (ppm)
OH
HO
N
O

(1) Hall, M. P.; Unch, J.; Binkowski, B. F.; Valley, M. P.; Butler, B. L.; Wood, M. G.; Otto, P.; Zimmerman, K.; Vidugiris, G.; MacHleidt, T.; et al. Engineered Luciferase Reporter from a Deep Sea Shrimp Utilizing a Novel Imidazopyrazinone Substrate. *ACS Chem. Biol.* **2012**, *7* (11), 1848–1857. https://doi.org/10.1021/cb3002478.

(2) Tannous, B. A.; Kim, D.; Fernandez, J. L.; Weissleder, R.; Breakefield, X. O. Codon-Optimized Gaussia Luciferase CDNA for Mammalian Gene Expression in Culture and in Vivo. *Mol. Ther.* **2005**, *11* (3), 435–443. https://doi.org/10.1016/j.ymthe.2004.10.016.

(3) Markova, S. V; Larionova, M. D.; Vysotski, E. S. Shining Light on the Secreted Luciferases of Marine Copepods : Current Knowledge and Applications. **2019**, No. 2, 705–721. https://doi.org/10.1111/php.13077.

(4) Shakhmin, A.; Hall, M. P.; Machleidt, T.; Walker, J. R.; Wood, V.; Kirkland, T. A. Biomolecular Chemistry Bioluminescence with NanoLuc †. **2017**, 8559–8567. https://doi.org/10.1039/c7ob01985h.

(5) Kierat, R. M.; Kra, R. A Fluorogenic and Chromogenic Probe That Detects the Esterase Activity of Trace Copper (II). *Bioorg. Med. Chem. Lett.* **2005**, *15*, 4824–4827. https://doi.org/10.1016/j.bmcl.2005.07.042.

(6) Yuan, M.; Ma, X.; Jiang, T.; Gao, Y.; Cui, Y.; Zhang, C.; Yang, X.; Huang, Y.; Du, L.; Yampolsky, I.; et al. Biomolecular Chemistry and Mice Using Novel Pro-Substrates for Renilla. **2017**, 10238–10244. https://doi.org/10.1039/c7ob01656e.

(7) Lindberg, E.; Mizukami, S.; Ibata, K.; Miyawaki, A. Development of Luminescent Coelenterazine Derivatives Activatable by b -Galactosidase for Monitoring Dual Gene Expression. *Chem. - A Eur. J.* **2013**, *19*, 14970–14976. https://doi.org/10.1002/chem.201302002.
